# Automotive Radar Processing With Spiking Neural Networks: Concepts and Challenges

**DOI:** 10.3389/fnins.2022.851774

**Published:** 2022-04-01

**Authors:** Bernhard Vogginger, Felix Kreutz, Javier López-Randulfe, Chen Liu, Robin Dietrich, Hector A. Gonzalez, Daniel Scholz, Nico Reeb, Daniel Auge, Julian Hille, Muhammad Arsalan, Florian Mirus, Cyprian Grassmann, Alois Knoll, Christian Mayr

**Affiliations:** ^1^Chair of Highly-Parallel VLSI-Systems and Neuro-Microelectronics, Faculty of Electrical and Computer Engineering, Institute of Principles of Electrical and Electronic Engineering, Technische Universität Dresden, Dresden, Germany; ^2^Infineon Technologies Dresden GmbH & Co., KG, Dresden, Germany; ^3^Department of Informatics, Technical University of Munich, Munich, Germany; ^4^Infineon Technologies AG, Munich, Germany; ^5^BMW Group, Research, New Technologies, Garching, Germany; ^6^Centre for Tactile Internet (CeTI) With Human-In-The-Loop, Cluster of Excellence, Technische Universität Dresden, Dresden, Germany

**Keywords:** spiking neural networks, FMCW, radar processing, MIMO, automotive, neuromorphic computing, signal processing

## Abstract

Frequency-modulated continuous wave radar sensors play an essential role for assisted and autonomous driving as they are robust under all weather and light conditions. However, the rising number of transmitters and receivers for obtaining a higher angular resolution increases the cost for digital signal processing. One promising approach for energy-efficient signal processing is the usage of brain-inspired spiking neural networks (SNNs) implemented on neuromorphic hardware. In this article we perform a step-by-step analysis of automotive radar processing and argue how spiking neural networks could replace or complement the conventional processing. We provide SNN examples for two processing steps and evaluate their accuracy and computational efficiency. For radar target detection, an SNN with temporal coding is competitive to the conventional approach at a low compute overhead. Instead, our SNN for target classification achieves an accuracy close to a reference artificial neural network while requiring 200 times less operations. Finally, we discuss the specific requirements and challenges for SNN-based radar processing on neuromorphic hardware. This study proves the general applicability of SNNs for automotive radar processing and sustains the prospect of energy-efficient realizations in automated vehicles.

## 1. Introduction

**Figure 1:**
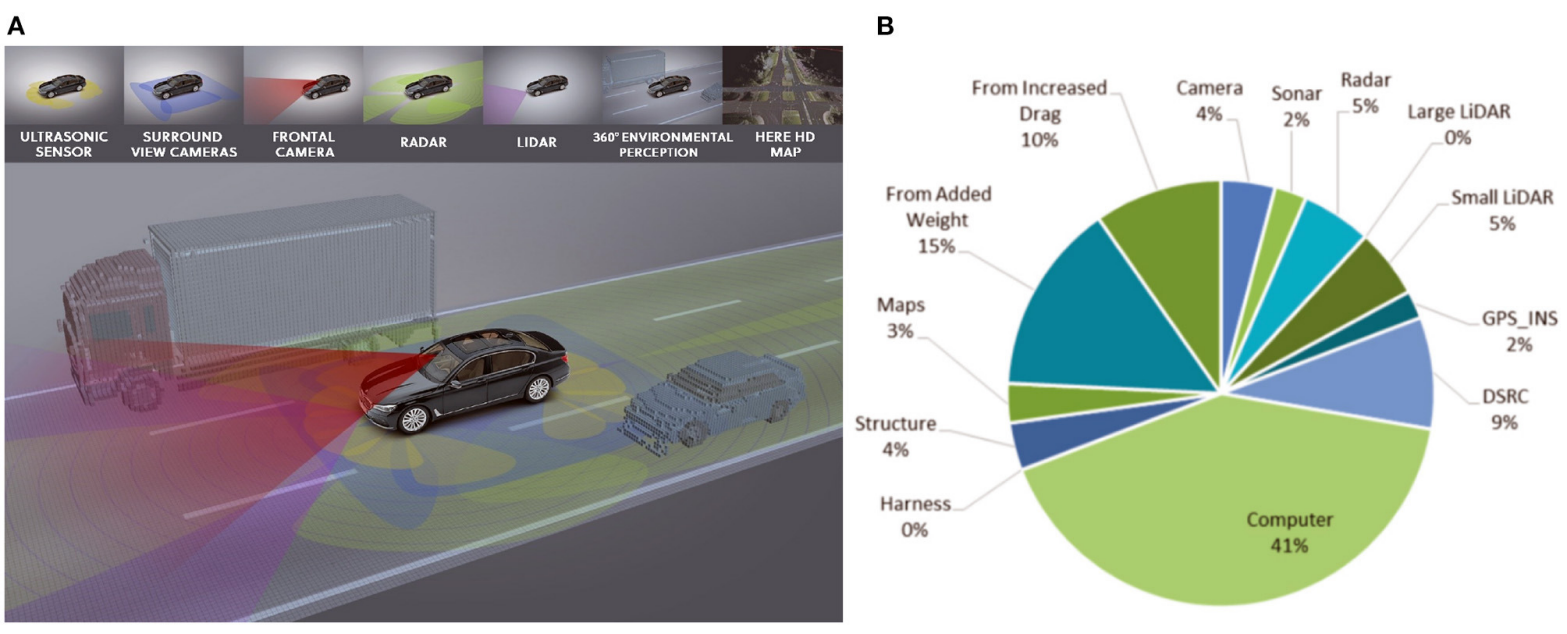
**(A)** Exemplary sensor setup of an automated vehicle prototype. Image source: BMW. **(B)** Sources of added energy consumption on a medium automated vehicle system on an electric vehicle prototype. Reprinted with permission from Gawron et al. (2018) Copyright 2018 American Chemical Society.

Automated driving is currently a very appealing area of research continuously drawing attention from academic and industrial research groups alike. One key aspect of this development is the success of modern machine learning approaches over the past decade, particularly deep learning by achieving remarkable results on several tasks necessary for fully automated driving, such as traffic sign recognition (Ciresan et al., 2012), semantic segmentation (Badrinarayanan et al., 2015), 2D and 3D object detection (Zhou et al., 2019; Yin et al., 2020), and behavior prediction of other traffic participants (Deo and Trivedi, 2018). Therefore, the use of such powerful learning approaches in automated vehicle functions and components is likely to increase in the near future. On the other hand, automated vehicle prototypes are typically equipped with a rich setup of various sensor units (Aeberhard et al., 2015, see also Figure 1A) to ensure a sufficient coverage of the vehicle's surroundings as well as safety through sensor redundancy. This combination of increasing in-vehicle deployment of modern and power-hungry machine learning approaches; rich and redundant sensor setups; and limited on-board energy resources poses significant challenges on the realization of automated vehicles: Already today, a significant amount of energy in automated vehicle prototypes is dedicated to computing (Gawron et al., 2018, see also Figure 1B). Furthermore, in electric vehicles high processing demands can significantly reduce the travel range. While the energy per operation in CPUs and GPUs decreases for smaller semiconductor manufacturing processes, researchers see an asymptotic efficiency wall that is slowly approached in the next years (Marr et al., 2013): Therefore, alternative approaches regarding hardware and algorithms are demanded that fulfill both the efficiency and safety requirements for autonomous vehicles.

The neuromorphic computing field (Roy et al., 2019) presents an attractive alternative to overcome the previously described challenges. It takes inspiration from the brain by means of a highly-parallel and local processing of information in neural networks, where the memory—the synaptic weights—is physically close to the computing units (neurons). Spiking neural networks (SNNs) employ event-based communication of information, which is fast, efficient and sparse, as information flows when something significant changes or happens. In turn, neuromorphic engineering (Mead, 1990; Indiveri et al., 2011) integrates neuro-inspired building blocks into electronic circuits for an energy-efficient sensing and information processing suitable for low-power edge applications or large-scale brain simulation. There exist several large-scale neuromorphic hardware systems for SNNs using either purely digital (Merolla et al., 2014; Davies et al., 2018), multi-processor based (Furber et al., 2014) or mixed-signal approaches (Qiao et al., 2015; Wunderlich et al., 2019) (see Furber, 2016; Thakur et al., 2018 for reviews). This is complemented with a new generation of sensors, such as dynamic vision sensors (Lichtsteiner et al., 2008; Brandli et al., 2014) or dynamic audio sensors (Liu et al., 2014), which enable a neuro-inspired pre-processing to directly output events, allowing a seamless integration to neuromorphic compute platforms. Still, those sensors and hardware platforms are mainly used in academic research and are just gradually making their way to commercial products, particularly in the automotive context.

In this article, as one step toward energy-efficient neuro-inspired processing for automated driving, we investigate the use of *spiking neural networks* for *automotive radar signal processing*. Automotive radars complement LIDAR sensors and cameras for the perception of the street scene and other road users. The used frequency modulated continuous wave (FMCW) radar sensors operate in the 77 GHz band and provide accurate range and relative velocity measurements for distances up to 250 m. In contrast to LIDAR and camera, automotive radar works reliably under all weather conditions and in scenarios with poor lighting, and it also achieves fast reaction times for automatic emergency breaking systems (Patole et al., 2017). However, traditional radars lack fine angular resolution to recognize and separate close targets in complex automotive scenarios, and to fully exploit their capabilities in the new artificial intelligent (AI) era. Recent research efforts (Khalid et al., 2018; Arkind et al., 2020; Rao et al., 2020) are tackling this problem by significantly increasing the number of transmit and receive antennas in a multiple input multiple output (MIMO) configuration, which enables a very high angular resolution (down to 1°). This new imaging radar trend has the potential to address the perception challenges in traditional automotive radar sensors, extend the detection to occluded situations in which a pedestrian is not yet exposed to the visual sensors, and provide an accurate radar-based classification of targets in all scenarios, which are all key aspects to enable fully automated driving.

Motivated by the successful application of SNNs for a wide range of signal processing and pattern recognition tasks (Zhou et al., 2020; Davies et al., 2021; Göltz et al., 2021; Yin et al., 2021), we want to explore whether the signal processing steps of automotive radars can be implemented with SNNs and how well those SNNs perform compared to conventional algorithms. To this end, we first collect and discuss SNN concepts for all steps of the radar processing chain. Next, in order to provide concrete examples, we implement and evaluate SNNs for two processing steps in software. Furthermore, as we plan a future implementation on digital neuromorphic hardware, such as Loihi (Davies et al., 2018) or SpiNNaker2 (Mayr et al., 2019), we derive the specific requirements and challenges of neuromorphic radar processing.

Our main contributions in this article are:

1. We perform a comprehensive analysis of the state-of-the-art digital signal processing (DSP) steps for automotive radars and discuss SNN-based approaches for all stages of the processing chain.
2. For the radar target detection step, we implement SNNs for two variants of the constant false alarm rate (CFAR) algorithm and compare their object detection performance and computational cost to classic approaches.
3. For the first time, we apply an SNN to automotive radar object classification achieving an accuracy close to a reference artificial neural network (ANN) at significantly reduced computational cost.
4. We derive the requirements for realizing SNN-based radar processing in neuromorphic hardware systems and discuss the encountered challenges.

The remainder of this article is organized as follows: Section 2 describes the operating principle of automotive radars and the digital signal processing chain. It further introduces spiking neural networks and the CARRADA automotive radar dataset used in this article. Section 3 presents a detailed assessment of SNN concepts with the potential to enhance or extend the previously described DSP chain. Section 4 implements and evaluates spiking neural networks for two radar processing steps. Finally, Section 5 discusses the challenges and future outlook in this direction.

## 2. Background

### 2.1. FMCW Radar

**Figure 2:**
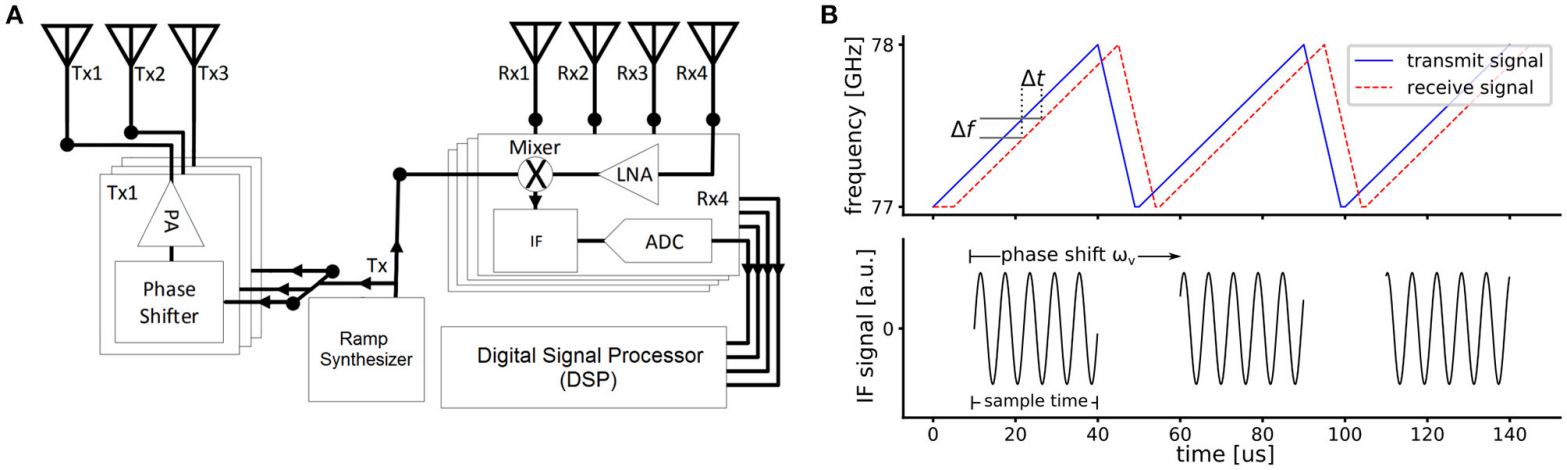
FMCW radar: **(A)** Schematic of radar frontend with 3 transmitters and 4 receivers. **(B)** FMCW radar principle showing a sequence of transmitted and received frequency chirps (top) and the sampled IF signal (bottom).

Frequency modulated continuous wave (FMCW) radar is massively used in cars for advanced driver assistance system (ADAS), and due to its robustness, it is considered an automotive industry standard. As its modulated waveform, it uses a continuous monotonic chirp, whose frequency increases (or decreases) linearly along its duration. Figure 2 shows a general block diagram of the FMCW radar, in which the reference signal (Tx) is generated in the ramp synthesizer, and transmitted *via* the antenna array (Tx1, Tx2, and Tx3) after its radiated power is increased using a power amplifier (PA). Each receiver block (Rx) mixes the Tx signal with the amplified target echo at the output of the low noise amplifier (LNA), and creates the intermediate frequency (IF) signal, which is digitized through the analog-to-digital converter (ADC). Considering a radar echo from a single object, the received frequency ramp will have a time shift Δ*t* proportional to the distance *d* to the radar sensor, which is equivalent to a frequency shift Δ*f*, as shown in Figure 2. After down-mixing the two signals, the reflection from a single radar object will contribute a sinusoid of frequency Δ*f* to the IF signal. This frequency is defined by:

(1) $$\begin{array}{r} \Delta f=\frac{2dB}{c_{0}T_{c}}, \end{array}$$

where *B* is the bandwidth of the chirp, *T*_*c*_ the chirp duration, and *c*_0_ the speed of light. In practice, the IF signal is a superposition of reflections from multiple targets with different Δ*f* and noise. The range of the targets can be extracted *via* the range-FFT (signal processing described in the Section 2.2).

Within a so-called radar frame, multiple of these fast chirps are transmitted successively to obtain the relative velocity: For an object that moves away from (toward) the sensor, the frequency shift Δ*f* increases (decreases) between chirps, although the shift is typically so small that it cannot be recognized after the range-FFT. Yet, the phase difference ω_*v*_ of the IF signal components between two consecutive chirps (cf. Figure 2B) contains the information about the relative velocity *v*:

(2) $$\begin{array}{r} v=\frac{\lambda \omega_{v}}{4\pi T_{c,diff}}, \end{array}$$

with the carrier wavelength λ (3.9 mm for 77 GHz radar) and the time between chirps *T*_*c,diff*_. To achieve a high accuracy for the velocity estimation, it is typically extracted by applying the so-called Doppler-FFT over all chirps within a frame (see, e.g., Patole et al., 2017 for further details).

In order to retrieve the angle of arrival (AoA) θ for one target, at least two receivers are needed. For an antenna array of two elements with a separation distance *d*, the reflected signal from the single target is captured with a phase difference (ω_θ_). Using far field approximation this phase difference can be calculated as

(3) $$\begin{array}{r} \omega_{\theta }=\frac{2\pi }{\lambda }d\sin\theta . \end{array}$$

By adding more receive and transmit antennas, the angular resolution for detecting target reflections and distinguishing them from other reflections can be increased. Typical automotive radar sensors have 3 transmitters and 4 receivers. While the receivers are arranged along the horizontal axis, the transmitters are arranged in an L-shape to also obtain an elevation angle (Sun et al., 2020). Hence, the so-called virtual antenna array in azimuth direction has 8 antennas. Yet, there is a trend to high-resolution radars with 64 antenna elements and above (Bilik et al., 2018; Och et al., 2018; Sun et al., 2020). The drawback of this MIMO approach is that it needs modulation schemes to ensure the separation of the individual contributions from each transmitter. The most used modulation scheme is time-division multiplexing (TDM), in which only one transmitter is enabled concurrently, but there are other approaches that use phase codes or frequency division multiplexing (Roos et al., 2019).

### 2.2. Radar Signal Processing

**Figure 3:**
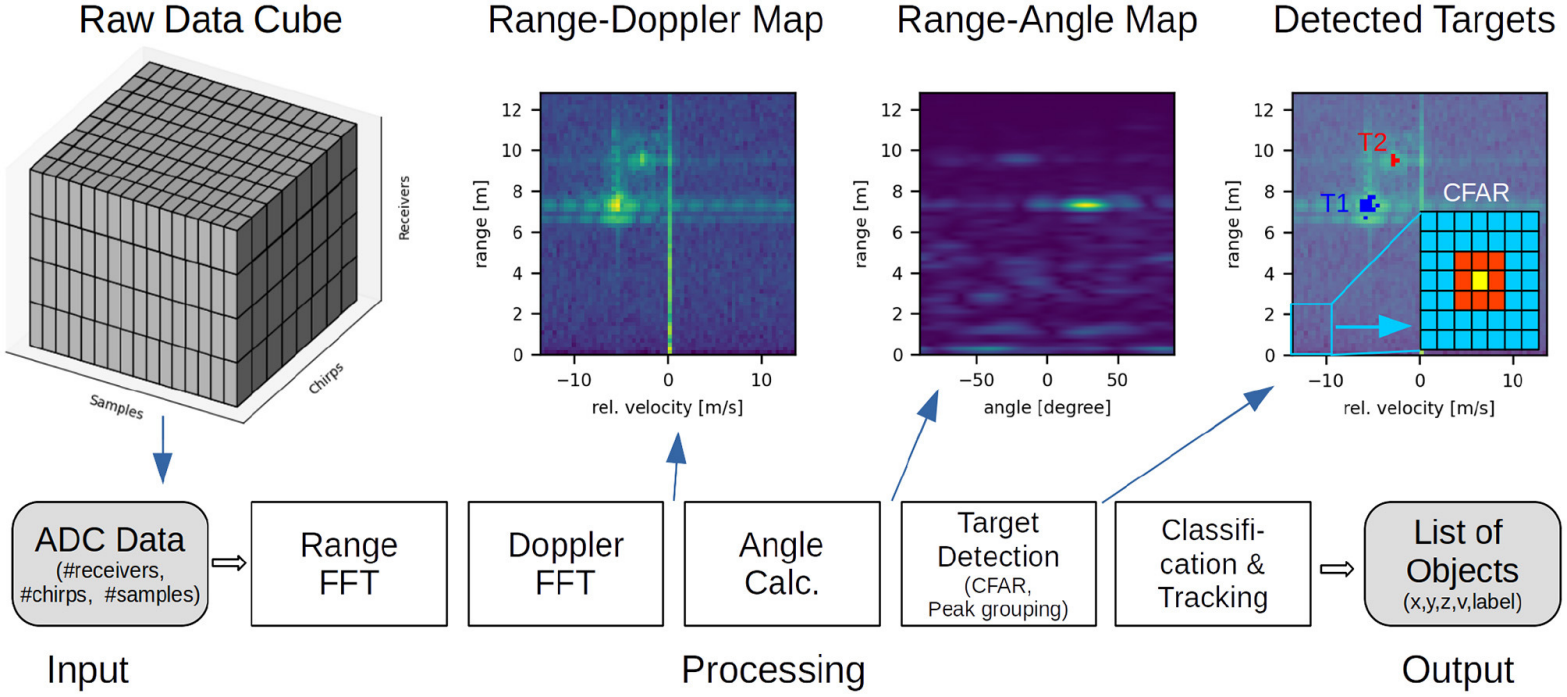
Conventional radar processing chain: The raw input data (ADC samples from multiple chirps and receivers) is processed by a sequence of algorithms yielding a list of detected objects with coordinates and labels. Intermediate data representations are shown in the top. In the top right figure, the inset shows the CFAR kernel for target detection with cell under test (yellow), guard cells (red), and training cells (blue).

In the following, we describe the steps for processing a single radar frame recorded with a MIMO FMCW sensor. The IF data recorded in a frame is organized as a data cube with 3 dimensions: the number of receivers *N*_RX_, the number of chirps per receiver *N*_chirps_, and the number of ADC samples per chirp *N*_samples_. A single sample is typically an integer value with 12 to 16 bits, or a complex number with two 16 bit integers in case of an IQ-baseband architecture (Ginsburg et al., 2018). Typical numbers for the three dimensions could be 4 receivers, 64 chirps, and 512 samples. In total, the complete raw data of one frame can require up to 256 KiB for the considered case of real-valued samples. The digital signal processing steps are illustrated in Figure 3, which are briefly described in the next sections. For further details see Patole et al. (2017) or Gamba (2020).

#### 2.2.1. Fourier Transform

The IF signal can be regarded as a superposition of sine waves with different frequencies and amplitudes corresponding to radar reflections from objects at different distances. The ADC samples additionally contain noise from radar clutter and the radar frontend.

##### 2.2.1.1. Range-FFT

The discrete Fourier transform (DFT) is applied on the IF samples of each chirp to obtain the frequency representation of the IF signal, which is related to the range of objects using Equation (1). As the fast Fourier transform (FFT) algorithm (Cooley and Tukey, 1965) is used for efficiency reasons, this step is called *range-FFT*. The output of such *N*-point FFT are *N* complex numbers representing the *N* frequency bins in the range $\left[-\frac{f_{s}}{2},\frac{f_{s}}{2}\right]$, where *f*_*s*_ is the ADC sampling rate. Typically, a window function like the Hann function is applied before the FFT computation to smooth the frequency response and reduce sidelobes in the frequency spectrum (Gamba, 2020, Section 3.7). In case of real-valued IF samples, the frequency spectrum is symmetric so that only the *N*/2 positive frequency bins are considered for the next processing steps.

##### 2.2.1.2. Doppler-FFT

The relative radial velocity of radar objects is obtained by applying a 2nd FFT on the output of the range-FFT across the chirps of a frame. The Doppler-FFT is applied individually for each range bin so that in total *N*/2 Doppler-FFTs are computed to generate a range-Doppler map for each receiver. In order to improve the SNR of the target, a systematic range-Doppler map is obtained by accumulating the range-Doppler maps from all *N*_RX_ receivers, which is shown in Figure 3. We note that, as the velocity calculation depends on the phase shift ω_*v*_ between two chirps Equation (2), there is a so-called maximum unambiguous velocity corresponding to ω_*v*_ = π. Larger relative velocities are mapped to the range [−π, π] and will appear at a negative or lower frequency bin in the Doppler spectrum. See Gonzalez et al. (2021) for more details and disambiguation techniques.

#### 2.2.2. Angle-of-Arrival Calculation

To obtain the angle-of-arrival, typically a Fourier transform is applied across the virtual antennas for each range-Doppler cell. Alternatively, there are more sophisticated approaches, such as MUSIC (Schmidt, 1986), or ESPRIT (Roy and Kailath, 1989). Still, the FFT is normally used due to the lower computational effort (Gentilho et al., 2019), and due to the existence of on-board FFT accelerators already available for the range and velocity calculation. The output of the angle calculation step can either be a range-Doppler-angle cube, or a range-angle map as illustrated in Figure 3. In addition to the primary azimuth direction, the elevation angle can also be computed depending on the antenna layout, providing a 3D ((*x, y, z*)) representation of the radar scene. Sometimes, the AoA calculation is postponed and only calculated for detected objects.

#### 2.2.3. Target Detection

The next task is to find and locate objects in processed radar data (range-Doppler map, range-angle map or radar-Doppler-angle cube). First, amplitude peaks are detected by an adaptive threshold mechanism. Second, detected peaks are clustered in groups belonging to the same object.

##### 2.2.3.1. Constant False Alarm Rate Algorithm

Radar spectra, such as the range-Doppler map, contain both target reflections and noise. The simplest approach to detect peaks is to compare them to a global threshold above the noise level. Such a threshold has to be chosen small enough to detect weak target reflections (e.g., distant pedestrians) but also high enough to avoid false alarms (noise detected as objects). As the noise and signal levels of the radar may vary depending on the signal source (range, angle) or weather conditions, an adaptive threshold is applied that aims to keep the false alarm rate constant. The so-called constant false alarm rate (CFAR) algorithm (Rohling, 1983) checks whether the amplitude of the cell under test (CUT) is significantly higher than the noise level *P*_noise_ of surrounding cells in the radar spectrum, e.g., a range-Doppler map:

(4) $$\begin{array}{r} x_{\text{CUT}}>\alpha P_{\text{noise}}. \end{array}$$

Here, α denotes a threshold factor that is related to the “constant false alarm rate,” which defines the desired rate of false object detections.

Common algorithms are the cell-averaging CFAR (CA-CFAR) which estimates *P*_noise_ as the average of the surrounding cells, and the ordered-statistic CFAR (OS-CFAR) which takes the *k*th largest value of the surround cells as noise estimate. In both cases, the so-called “guard cells” close to the CUT are discarded for noise estimation, as they may contain reflections from the same radar object (see Figure 3 for an illustration of the CFAR kernel in a range-Doppler map).

##### 2.2.3.2. Clustering/Peak Grouping

Clustering algorithms are in charge of grouping the sparse point clouds provided by the object detection stage into blobs that represent the different objects in the scene. In other words, the clustering stage assigns a label to each point, where each label identifies a unique object. The points that correspond to noise can either be left unlabeled or be assigned to a dummy label. In Figure 3, the detected reflection points are clustered in two targets (T1, T2) with different colors.

Clustering algorithms are generally divided into partitioning algorithms, where the amount of clusters is decided beforehand, and hierarchical algorithms, which organize clusters in a tree-structure with an undetermined number of nodes. Even though the former offer higher computational and memory efficiency, they are not adequate for the automotive radar processing as cars typically navigate through unknown scenarios with a dynamic number of objects around them.

Perhaps the most popular hierarchical clustering algorithm is DBSCAN (Density-based spatial clustering of applications with noise, Ester et al., 1996). First, the density around each point *p* is computed. Then, all points with density higher than an arbitrary threshold are considered core-points. Finally, all core points that are density-reachable are clustered together.

Another clustering algorithm with similar complexity is DENCLUE (Hinneburg et al., 1998). Similar to DBSCAN, DENCLUE creates a density map of the input space. However, the latter calculates the density gradient afterwards and performs a hill-climbing procedure for connecting points that can be connected by a low-gradient path. When comparing both, DENCLUE shows small benefits in terms of efficiency, but it involves a more complicated tuning that makes it harder to be generalized for changing environments.

#### 2.2.4. Target Classification

The next step in the radar processing chain is the classification of the detected radar objects into categories, such as vehicles, pedestrians, cyclists, buildings, or traffic signs. The classical approach for target recognition is to identify features for the radar data and then apply a machine learning classifier such as a support vector machine (SVM) (Heuel and Rohling, 2011, 2012; Lee et al., 2017). In this case, the features used for classification are typically hand-crafted and include primary parameters, such as range and velocity but also the radar cross section (RCS) or the extension of detected clusters (Bartsch et al., 2012). Subsequently, supervised learning is used to train a classifier. While these approaches are effective and computationally efficient, they do require expert knowledge for feature extraction. Furthermore, the usability of the features may be limited to a specific problem or dataset.

Most recent approaches therefore rely on deep neural networks (DNNs) for radar object classification, since they do not require manual feature selection and extraction. These approaches can be further divided into those using convolutional neural networks (CNNs) (Kim and Moon, 2016; Schumann et al., 2017; Capobianco et al., 2018; Patel et al., 2019; Pérez et al., 2019), recurrent neural network (RNN) (Klarenbeek et al., 2017; Schumann et al., 2017) or a combination of both (Angelov et al., 2018; Kim et al., 2018). Most approaches process the range-Doppler map, while the majority of those focusing on moving target classification are based on micro-Doppler signatures. A few approaches also make use of additionally processed radar data for classification. In Meyer and Kuschk (2019b), the authors fuse the information from a 3D radar point cloud with camera data for object detection. Schumann et al. (2017) cluster the points and combine them with a number of features for classification with an LSTM and a random forest algorithm. On the other contrary, Patel et al. (2019) process the range-angle map for target classification: A region of interest (ROI) of fixed size around the center of each detected object is classified with a 3-layer CNN into seven different object types.

#### 2.2.5. Target Tracking

Tracking the movement of road users is essential for automated driving as it allows to predict future trajectories. A common approach for tracking single radar targets is the Kalman filter (Kalman, 1960), that iteratively optimizes its parameters from noisy observations to predict the next system state (*x, y, z*, and the velocity vector of radar target). Often, the extended Kalman filter is used as it allows to predict position and velocity in Cartesian coordinates from observations of range and angles of arrival (Ikram and Ali, 2013). Other methods like Bayesian filtering can also be applied to radar object tracking (Gordon et al., 1993).

In case of multiple objects in the radar scene, there is a data association problem, as the detected objects in each frame need to be assigned to tracks. Radar targets may appear or disappear from the radar field of view so that new tracks have to be created and old ones deleted. The algorithms should also be able to track objects that are temporarily occluded, such as small pedestrians behind parking cars. Common approaches for data association are the rather simple generalized nearest neighbor (GNN) algorithm that minimizes the distance between tracks and detections, and the more compute-intensive joint probabilistic data association (JPDA). We refer to (Gamba, 2020, Section 7.4) for further information.

### 2.3. Spiking Neural Networks

#### 2.3.1. Spiking Neurons

**Figure 4:**
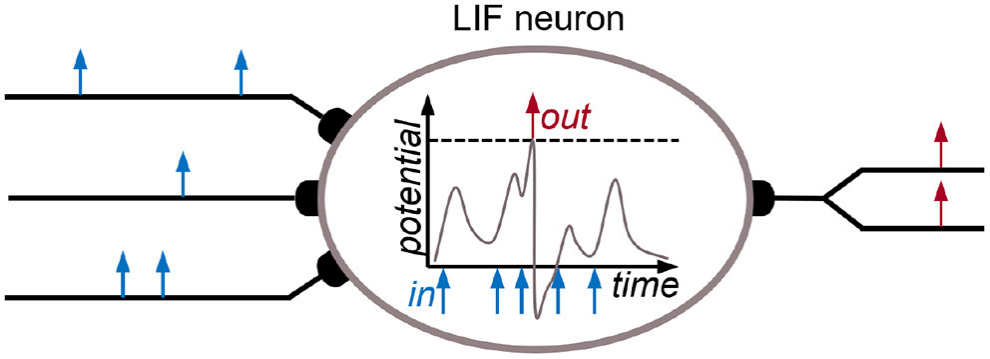
Schematic illustration of a leaky integrate & fire (LIF) neuron, where multiple spikes (blue) from different input neurons lead to an output spike (red) of the given neuron. In the center, the course of the membrane potential over time is shown: When reaching the spike threshold (dashed line), the potential is reset and a spike is sent out to other neurons.

Spiking neurons are a subclass of artificial neurons that communicate *via* spike events with each other. These neurons typically have an internal state, that is called membrane potential, inspired from biological neurons. Whenever the membrane potential reaches a certain threshold, its value is reset and a spike is sent to all connected neurons. At the target neurons, the spike leads to a change of the membrane potential dependent on the strength of the connection – the so-called synaptic weight. This process is illustrated in Figure 4. In contrast to artificial neurons, which continuously forward scalar values to their connected neurons, SNNs convey information in the timing and count of spikes. Technically, SNNs resemble artificial RNNs as the neurons have states, i.e., the membrane potential. Therefore, SNNs are considered candidates for efficient and effective processing of spatio-temporal data. Two very common neuron models are the integrate & fire (I&F) neuron, which integrates incoming synaptic events and resets the membrane voltage after reaching its threshold, and the leaky integrate & fire (LIF) neuron, whose membrane potential decays over time. Spiking neurons can be connected in a pure feed-forward fashion, where each layer encodes some features which are then forwarded to the next layer. However, spiking networks achieve their optimum efficiency with more complex network structures, such as combinations of recurrent and feed-forward connections (Yin et al., 2021).

#### 2.3.2. Neural Codes

Here we summarize common spike coding mechanisms that have potential for radar processing with SNNs. For all cases, we need to distinguish between *encoding*, which means the conversion of arbitrary input into spikes, and *decoding*, the extraction of results from spike data. Both may be applied for single or multiple neurons. For encoding one may further differentiate between one-time inputs (e.g., a gray-scale value of an image pixel) and time-varying signals such as an ECG signal.

*Rate coding* translates a scalar input value into the firing rate of an associated spike source. Spikes are either generated with a fixed interval or in Poisson neurons with random spike times according to a given firing probability. As spike rates can only be positive, signed input values need to be either scaled and shifted to positive spike rates or represented by two spike sources representing positive and negative values, respectively. To decode information from spike trains, the number of spikes has to be counted and averaged over a certain time window. Rate codes typically require many spikes and long simulation times for an accurate encoding and are thus rather computationally expensive.

In contrast, *temporal codes* use the spike timing to carry information. The *latency* or *time-to-first-spike* code translates input values to single spikes per source neuron where typically higher values are mapped to lower spike times. Similarly, the timing of output spikes of a network can be used to extract results, e.g., the neuron with the first spike predicts the class of an image (Mostafa, 2017). Another temporal approach is *rank-order coding* (Thorpe and Gautrais, 1998), where the order of spikes from different neurons encodes information. In contrast to the latency code, the exact spike times do not matter and no external reference such as the start time is needed. Similarly, in *phase coding* an internal oscillatory signal like the gamma waves may provide a reference signal for temporal codes. Temporal codes are computationally more efficient than rate codes as they require less spikes, yet one challenge is to achieve a high temporal precision in simulation or emulation on neuromorphic hardware.

There are many more approaches for spike encoding, including *delta encoding* as applied in dynamic vision sensors that output ON or OFF events when the input intensity changes. The *current injection* approach modifies the input current to an LIF or IF neuron; *population coding* uses multiple neurons for value representation and unconventional approaches may combine several of the above mentioned concepts (Schuman et al., 2019). For a survey of encoding techniques (see Auge et al., 2021b).

#### 2.3.3. Network Architectures and Training

SNNs theoretically exhibit extraordinary computational power (Maass, 1997), yet not many approaches exist that demonstrate this ability in practice. One way to approximate dedicated functions is to *construct* networks from scratch including connectivity, weights, neuron models and parameters. Common general approaches for that are the neural engineering framework (Eliasmith and Anderson, 2003) or liquid state machines (Maass et al., 2002). Besides, one can take inspiration and re-use networks, connection motifs, and principles from biology such as receptive fields as filters in the visual pathway or winner-take-all networks as two examples.

Regarding network *training* the brain offers unsupervised mechanisms such as Hebbian learning or spike-timing-dependent plasticity (STDP) (Bi and Poo, 1998) to adapt weights based on pre-and postsynaptic activity. This for example allows neurons to specialize on certain spatio-temporal features of the input (Masquelier et al., 2008). Reward-based learning is realized by adding neuromodulation to synaptic plasticity (Frémaux and Gerstner, 2016). For supervised learning, as applied to deep neural networks with the error backpropagation, there is no direct equivalent for SNNs due to the discontinuity of the membrane voltage after spiking leading to a non-differentiability. Yet, in the last years many approaches have been developed to create deep spiking networks with similar performance as DNNs for image classification, either by conversion (Rueckauer et al., 2017; Sengupta et al., 2019) or direct training, e.g., using surrogate gradients as an approximation mechanism (Wu et al., 2018; Zenke and Ganguli, 2018). Recent work has shown that recurrent spiking networks can also be trained to high accuracy for sequential data using backpropagation through time (BPTT) with surrogate gradients (Neftci et al., 2019; Yin et al., 2021) or more bio-inspired approaches like e-prop (Bellec et al., 2020).

### 2.4. CARRADA Dataset

The recently published CARRADA dataset (Ouaknine et al., 2020) is one out of few publicly available automotive datasets containing not only vision and LIDAR/depth information but also radar data. Most datasets do not include radar data at all (Geiger et al., 2013; Yu et al., 2020), but even if they do, the radar data included is usually in form of point cloud information (Caesar et al., 2019; Meyer and Kuschk, 2019a; Schumann et al., 2021), providing the (*x, y, z*) coordinates and the relative velocity of objects. The CARRADA dataset, on the other hand, includes the range-Doppler as well as the range-angle map for each scan. Still, it is limited in size, complexity and variety compared to the aforementioned datasets, as it is recorded on a remote test track in Canada with low environmental noise.

The CARRADA dataset consists of 30 separate sequences with a mean number of 422 frames per sequence (0.7 min) gathered from a synchronized setup composed of an FMCW radar and a camera mounted on a stationary car. Out of the total 12666 frames taken, 7,193 are annotated, containing one or two moving objects (car, pedestrian or cyclist). Each frame contains 3 different annotations (bounding boxes, sparse points and dense masks), making the dataset suitable for different tasks like object detection, semantic segmentation or tracking. The experiments presented in Section 4 make use of this dataset.

## 3. Radar Processing With SNNs: Concepts

In this section, we discuss concepts for replacing radar processing steps with SNNs. For each step, we review common spiking network architectures and principles of information processing in the brain that potentially can replace the conventional algorithms. Here, we mainly seek for SNNs that can solve single steps. How to combine SNNs to realize the complete processing chain, e.g., how to use the output spikes from on step as the input spikes to the SNN of the next step, is not covered here. We consider this overview of concepts an initial collection that inspires the use of SNNs for radar processing, but not claim for completeness.

### 3.1. Fourier Transform

The Fourier transform is typically applied in three different dimensions in automotive radar applications, i.e., the range, angle, and velocity. While the efficiency of the FFT algorithm is unquestionable, we consider SNNs for frequency spectrum analysis as they might be implemented very efficiently on neuromorphic hardware: We first discuss the use of resonate & fire (RF) neurons, continue with a recent spiking realization of the discrete Fourier transform and conclude with other brain-inspired approaches.

#### 3.1.1. Resonate-and-Fire Neurons

The RF neuron (Izhikevich, 2001) is a two-dimensional neuron model that shows oscillatory dynamics depending on its input. Here, the two coupled state variables $x=\left[\begin{array}{c} x_{1}\\ x_{2} \end{array}\right]$ of each neuron resonate with their Eigen frequency ω_0_ if the associated spectral component is present in the signal. The signal itself is directly fed into the neurons as the current *I*:

(5) $$\begin{array}{r} \dot{x}=\left[\begin{array}{cc} -d & -\omega_{0}\\ \omega_{0} & -d \end{array}\right]x+\left[\begin{array}{c} I\\ 0 \end{array}\right] \end{array}$$

Additionally, a damping constant *d* controls the resonance behavior of the neurons. A spike is generated as soon as the second variable *x*_2_ reaches the firing threshold. The spike pattern of an RF neuron contains information about the frequency, amplitude, phase, and their temporal development in the analyzed signal (Auge et al., 2021a).

For radar processing, the straightforward approach is to feed the IF signal as input *I* to an array of RF neurons with different resonant frequencies. The amplitude of the spectral component of the signal directly translates to the firing time of the neuron with the associated resonant frequency. The phase ϕ of the signal leads to an additional but much smaller shift of the spike time $\Delta t=\frac{\phi }{\omega_{0}}$. However, this phased-based time shift is much smaller than spike time variations introduced by noise in the input signal (Auge and Mueller, 2020). As for both range-Doppler analysis and angle estimation a high phase accuracy in the presence of noise is required, RF neurons are not suited for the present application. Still, the power density spectrum of the signal can be used in applications which do not rely on accurate phase estimations. We remark that the RF neuron model in Equation (5) has been recently implemented in the Loihi2 chip for audio processing (Orchard et al., 2021).

#### 3.1.2. Spiking Discrete Fourier Transform

We have proposed another alternative that replicates the Fourier transform (FT) calculation by using a non-leaky I&F spiking model (López-Randulfe et al., 2022). The architecture and weights of this model are derived from the trigonometric equation of the discrete Fourier transform,

(6) $$\begin{array}{r} Y_{k}=\overset{L-1}{\underset{l=0}{\sum }}X_{l}\left[cos\left(\frac{2\pi }{L}kl\right)-i\cdot sin\left(\frac{2\pi }{L}kl\right)\right]. \end{array}$$

where *Y*_*k*_ is the output of the *k*th frequency bin and *L* is the size of the input vector *X*. The previous equation can be rewritten for the *n*th FT dimension as the algebraic linear system

(7) [Re(Y(n))Im(Y(n))]=[WReWIm-WImWRe][Re(Y(n-1))TIm(Y(n-1))T],

which can be implemented as a neural layer with 2 × *L* neurons, where half of them represent the real values of the DFT and the other half represent the imaginary values, and *W*_Re_ and *W*_Im_ are derived from Equation (6). The spiking Fourier transform (S-FT) network applies time coding for computing the FFT: Inputs are represented by spiking neurons with a single spike at a time inversely proportional to the respective input values *X*_*l*_. The neuron model is able to accurately reproduce vector-matrix multiplications by splitting the operation in two stages. In the first stage, called silent stage, the neuron accumulates information from all pre-synaptic connections without producing a spike. In a second stage, the neuron is charged with a constant current and the output values are obtained from the firing times of the I&F neurons at the output. The experiments on the S-FT have tested its output error, energy consumption, and execution time for an implementation in the neuromorphic chip Loihi.

#### 3.1.3. Other Approaches

Other works in recent years proposed spiking networks for doing partial or full analysis of the frequency spectrum of temporal signals. In Jiménez-Fernández et al. (2016), the authors explored the usage of SNNs for extracting specific frequencies from silicon cochleas, i.e., neuromorphic implementations of the cochlea that output spikes (Chan et al., 2007).

The authors in Sabatier et al. (2017) suggest an asynchronous event-driven Fourier analysis that triggers an update of the DFT outputs only when an input value changes more than a predefined significance threshold. Note that the approach uses events with scalar values and not spikes. The algorithm is applied for the Fourier analysis of data from an event-based vision sensor: As the light intensity of pixels changes rather slowly, a high reduction of computations is demonstrated. The applicability to FMCW radar is limited as the first FT is applied to the time-varying IF signal which changes at high frequency. Yet, applying this approach to the Doppler or Angle-FFT seems more suitable as their input values generally change slowly.

Also noteworthy are principles from the brain, where neurons develop spectrotemporal receptive fields (see, e.g., Theunissen and Elie, 2014) and thus can specialize for specific input patterns. Yet, it seems challenging to transfer this to FMCW radar, as there are two time dimensions (so-called “fast time” for range and “slow time” for velocity extraction). Any approach would be further complicated by the underlying MIMO coding schemes (Section 2.1).

### 3.2. Angle-of-Arrival Calculation

In addition to replacing the angle FFT with a spiking neural network, we discuss other approaches for angle calculation: Looking at the brain, this problem resembles the sound localization which uses interaural time differences (ITD) for the AoA computation. Highly experienced echo-locators such as bats employ interaural level differences (ILD) instead, which in contrast to the ITD using their small heads, allows them to capture a wide diversity of target cross-sections at different ranges by sensing pressure differences across their ears. Engineering ITD methods require the concept of phase locking and delay lines so that certain neurons show a high firing rate when a certain frequency arrives at a certain AoA (Carr and Konishi, 1990). The concept has been proven in neuromorphic hardware with spiking neurons (Pfeil et al., 2013). However, it seems challenging or even unrealistic to apply the ITD or ILD methods to radar processing: For the continuous wave radar approach, there are no time differences measurable at different receivers, also the phase shifts are very small and would need to be pre-processed to act as an input to a neural network based on ITD or ILD. More complexity is added as there is not a single transmitter, but there are multiple that alternate in being active such that input data would need to be buffered before being processed as a larger virtual receiver array.

As conclusion of our analysis, the spiking Fourier transform from Section 3.1.2 seems to be the only suitable approach for the angle-of-arrival calculation so far. Yet, further research should be carried out on replacing high-resolution algorithms such as MUSIC or ESPRIT.

### 3.3. Target Detection

The classical approach uses the constant false alarm rate algorithm to adjust a local threshold to distinguish radar object reflections from noise. In a second step, the reflections are assigned or grouped to clusters representing the same radar object. We present two constructed SNNs implementing two different CFAR algorithms and briefly discuss spiking network approaches for clustering and grouping.

#### 3.3.1. Spiking OS-CFAR

The OS-CFAR algorithm is one of the most popular algorithms for object detection in radar data, which uses the *k*th largest value of the surrounding cells as noise estimate *P*_noise_ (Equation (4)). Due to the required sorting of neighbor values, it was termed order-statistic CFAR (Rohling, 1983).

**Figure 5:**
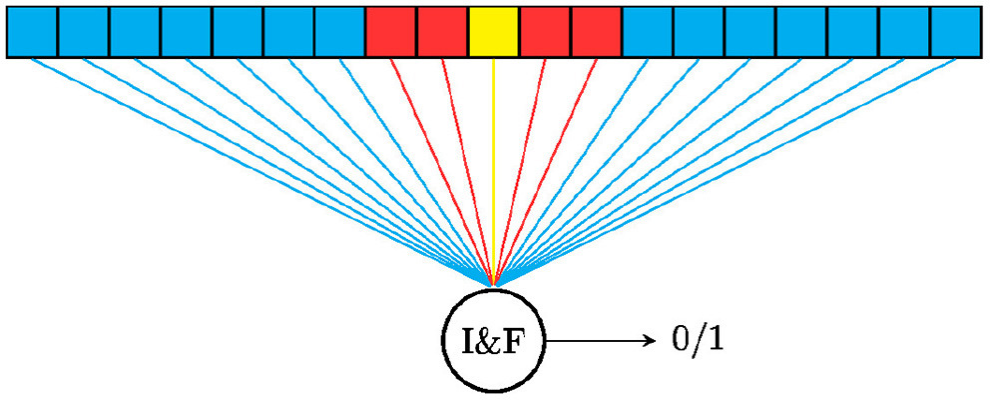
Diagram of the spiking CFAR approaches for one cell. The cell under test is shown in yellow, the red cells are guard cells and have no influence on the result, and the blue cells are the neighbor elements, also called training cells. The weights are set differently for the spiking OS-CFAR and spiking CA-CFAR. Figure redrawn from López-Randulfe et al. (2021).

In recent work, we have designed an SNN that approximates the OS-CFAR by using a one-layer network that takes as input temporal-coded spikes (López-Randulfe et al., 2021). All neighbor cells are connected with the same negative weight −*w*_*N*_, and the value under consideration is connected with a positive weight *kw*_*c*_. Therefore, the output neuron will produce a spike if and only if the CUT spikes before *k* neighboring neurons. Figure 5 shows the connection scheme of this network for a single cell in the input map.

#### 3.3.2. Spiking CA-CFAR

Another common approach to discern object reflections from noise is the cell-averaging CFAR Rohling (1983), which computes the noise level as average of *N* training cells:

(8) $$\begin{array}{r} P_{\text{noise}}=\frac{1}{N}\overset{N}{\underset{i=1}{\sum }}x_{\text{train},i}. \end{array}$$

In the following, we propose a spiking network that implements the CA-CFAR exactly using temporal coding. The CFAR condition *x*_CUT_ > α*P*_noise_ Equation (4) can be rewritten by means of a dot product of the vectors $\hat{x}$ and ***w***:

(9) $$\begin{array}{r} \hat{x}\cdot w>0, \end{array}$$

with $\hat{x}:=\langle x_{\text{CUT}},x_{\text{train},1},...,x_{\text{train},\text{N}}\rangle$ and $w:=\langle 1,-\frac{\alpha }{N},...,-\frac{\alpha }{N}\rangle$.

Equation (9) is equivalent to an artificial neuron with inputs $\hat{x}$, weights ***w*** and the Heaviside step function as nonlinearity. The same behavior can be realized with an integrate-and-fire neuron with current input and latency coding of input spikes. The input values ${\hat{x}}_{i}$ are translated into spike times *t*_*i*_ with a fixed linear mapping to an interval [0, *T*]:

(10) $$\begin{array}{r} t_{i}\leftarrow \frac{{\hat{x}}_{\text{max}}-{\hat{x}}_{i}}{{\hat{x}}_{\text{max}}}\cdot T, \end{array}$$

where ${\hat{x}}_{\text{max}}$ is an upper bound on all input values. The higher the input value, the earlier the spike time. The neuron equation is defined as:

(11) $$\begin{array}{r} I\left(t\right)=\underset{i}{\sum }w_{i}\Theta \left(t-t_{i}\right), \end{array}$$

(12) $$\begin{array}{r} \frac{dv}{dt}=I, \end{array}$$

where Θ(·) is the Heaviside step function. In Equation (11), for each input spike *i*, the current *I* is increased by the weight *w*_*i*_ at time *t*_*i*_. After the neuron is simulated for duration *T*, it is checked whether the voltage *v* is positive. If this is the case, the CUT fulfills the CFAR condition and generates a spike. In practice, each product ${\hat{x}}_{i}\cdot w_{i}$ in Equation (9) is emulated by the integral of its contribution to the current *I*, whose amplitude is *w*_*i*_ during the time [*t*_*i*_, *T*] and zero before (see Supplementary Section 1.2.1 for the proof of mathematical equivalence to the original CA-CFAR).

Both spiking CFAR algorithms are evaluated on the CARRADA dataset in Section 4.1.

#### 3.3.3. Clustering/Peak Grouping

There are several different approaches one could implement and evaluate for the clustering of reflections in the range-Doppler or range-angle maps. They can be divided into three overall categories: clustering with radial basis function (RBF) networks, (continuous) attractor networks, and CNNs.

There are a number of spiking clustering approaches which are based on the concept of spiking RBF neurons, introduced originally by Hopfield (1995) for pattern recognition. Natschläger and Ruf (1998) and Bohte et al. (2002) extend and evaluate this approach by, e.g., increasing the scalability. All approaches are using temporal coding for the input values with one input neuron for each dimension in the basic case. The clustering is performed by updating the weights for multiple, differently delayed synapses between the input and RBF neurons so that each RBF neuron spikes maximally for a single cluster. The weights are trained in an unsupervised manner using a Hebbian learning rule. A network here consists of *n* input neurons, one for each dimension of the input data, *m* RBF neurons, one for each cluster, and *l* synapses between each input neuron and each RBF neuron, depending on the discretization/granularity of the data.

The so-called SpikeCD approach by Lin et al. (2019) uses a clustering degeneracy algorithm with RBF neurons in order to dynamically adjust the number of clusters in the network. The performance is further improved by a supervised learning algorithm and the system is evaluated on multiple complex clustering tasks. SpikeCD overcomes the performance and parameterization issues of the classic RBF networks. Furthermore, the authors introduce a supervised classification to the clustering network. A similar setup could be used not only to cluster the data from, e.g., a range-angle map but also add a subsequent classification of the clustered points. Frady et al. (2020) have already demonstrated, that a spiking implementation of the *k*-NN algorithm on neuromorphic hardware (Loihi) is able to solve large scale clustering tasks with superior latency while being more energy efficient than traditional CPU-based algorithms. Diamond et al. (2019) performed similar experiments with their unsupervised spiking clustering algorithm but on the SpiNNaker platform.

One of the major disadvantages of the RBF neuron based clustering approaches is that each point from, e.g., a range-Doppler map needs to be processed individually and even multiple times, in order for the network to settle to a stable cluster. A similar functionality can be also realized with continuous winner-take-all attractor networks of spiking neurons, with one neuron for each data point in the range-Doppler map and a Mexican-hat like connection structure (Vogels et al., 2005). The synapses in this network would be excitatory to nearby neurons and inhibitory to those further away. A network with such an architecture is generally able to process the whole range-Doppler map at once, while possibly needing some time to settle into a stable state.

Object detection and localization is also performed in the visual cortex in the ventral and dorsal stream (Desimone and Duncan, 1995). Artificial neural networks like CNNs have taken inspiration from that and are now highly-performant for this task (Ebrahimpour et al., 2019). In such approach, several radar processing steps (target detection, clustering and classification) can be realized by a single artificial neural network as demonstrated by Pérez et al. (2019). Given the successful conversion of the popular YOLO model (Redmon et al., 2016) for object detection in images to a spiking network (Kim et al., 2020), we expect that a similar translation is also possible for the automotive radar domain.

### 3.4. Target Classification

The state-of-the-art approaches for target classification mostly use ANNs like CNNs or RNNs (Section 2.2.4). As mentioned in Section 2.3.3, SNNs for image and sequence classification can be obtained by conversion from DNNs or direct training. In the following, we focus on the classification of single radar objects with SNNs, i.e., we expect that only a single target is present in the input data. This can be achieved by extracting ROIs from the radar data making use of the clustered object reflections from the previous processing step.

To the best of our knowledge, so far SNNs have not been applied to *automotive radar object classification*, yet there is a variety of work on *radar gesture recognition* using SNNs which differ in the coding of the input data and network architectures: For the SoLi dataset (Wang et al., 2016), which provides sequences of range-Doppler maps, Yin et al. (2021) trained a network of several recurrent SNN layers with adaptive spiking neurons using surrogate gradients and BPTT. Similarly, Safa et al. (2021a,b) trained a spiking convolutional network achieving a higher accuracy. Both approaches turn the range-Doppler maps to spikes by thresholding. Instead, Tsang et al. (2021) feeds the spiketrains into a liquid state machine, a recurrent network of spiking neurons retaining a memory of received input, and evaluates various classifiers as read-out: Using an SVM a state-of-the-art accuracy for SoLi of greater than 98% is reached, which is superior to any DNN approach. For a non-public radar gesture dataset, in Kreutz et al. (2021) we combine the AoA information with range-Doppler maps from multiple frames to train deep SNNs with surrogate gradients and temporal coding. Different ways of encoding scalar values into spikes are evaluated.

Other SNNs operate on the micro-Doppler patterns: For the IMEC 8GHz dataset, Stuijt et al. (2021) treat the micro-Doppler as a binary image, train a DNN and convert it to a rate-based SNN. For the same dataset, Safa et al. (2021b) improve the classification accuracy by means of time-to-first-spike coding, a direct training of the spiking CNN and further preprocessing. Instead, in Arsalan et al. (2021), we treat the micro-Doppler pattern as a sequence of velocity vectors which is then fed into a SNN consisting of a 1D convolution layer, one dense LIF hidden layer, and an output layer. The network is trained with a SoftLIF (Hunsberger and Eliasmith, 2016) activation (an approximation to LIF) in the NengoDL framework (Rasmussen, 2019). Note that here the conversion to spikes only happens after the first convolutional layer. In a very different approach Banerjee et al. (2020) apply unsupervised learning (STDP) to train the weights of spiking convolution layers on binarized micro-Doppler sequences. A logistic-regression-based classifier acts on the output of all spiking convolutional layers.

In this work, we combine several of the concepts from radar gesture recognition for automotive radar object classification: Sequences of ROIs in the range-Doppler maps are classified with a spiking convolutional network with recurrent layers that was trained using surrogate gradients and BPTT. For details see Section 4.2.

### 3.5. Target Tracking

Neurological experiments with different types of mammals have shown, that they do some kind of path integration, i.e., they are able to infer their current position relative to some reference point with the help of e.g., locomotion signals (Etienne and Jeffery, 2004). (Continuous) attractor networks have not only a high degree of biological plausibility but also have been the most successful network type for modeling path integration (Redish and Touretzky, 1997). These networks have already been deployed successfully to real-world problems like mobile robot localization and mapping (Milford et al., 2004), as they are capable of keeping a (Gaussian) state representation continuously, even in the absence of any input.

Since the problem of path integration, which is a subproblem of simultaneous localization and mapping (SLAM), is very similar to the tracking of targets, we expect that this approach can be adapted for tracking objects in range-Doppler or range-angle maps. We further assume that with some changes in the connections and weights of the network it is possible to implement a clustering algorithm, similar to the ones used for clustering the data points in a range-Doppler map.

In such case, both the clustering and tracking could be solved by the same network(cf. Section 3.3.3).

## 4. Radar Processing With SNNs: Examples

In Section 3, we have presented *concepts* for SNN-based radar processing. Here, we provide *examples* of SNNs for solving two of the radar processing steps: target detection and target classification. Beyond demonstrating a proof-of-concept, the solutions are compared to the conventional state-of-the-art approaches considering also the limitations when SNNs are processed on hardware.

### 4.1. Target Detection With Spiking CFAR Algorithms

In Section 3.3, we have presented two SNNs that use temporal encoding for replacing the CA-CFAR and OS-CFAR algorithms, respectively. While both of them are mathematically equivalent to the original algorithms, their performance may deteriorate when being realized on neuromorphic hardware due to limited parameter resolution or discretization of spike times.

**Figure 6:**
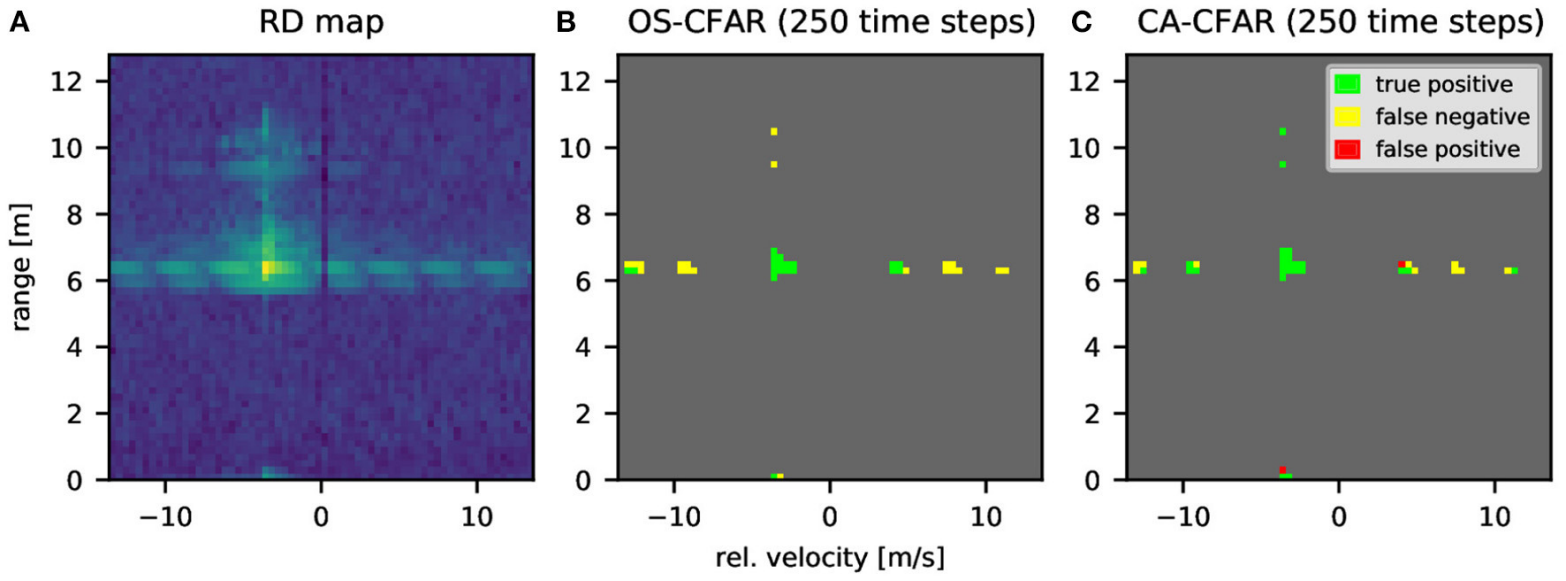
Example of object detection with spiking CFAR algorithms. **(A)** Exemplary range-Doppler map from CARRADA dataset. **(B,C)** Results of spiking OS-CFAR **(B)** and spiking CA-CFAR **(C)** applied to the range-Doppler map from A and comparison to original algorithms. Green points mark reflections detected by both classical and spiking algorithm. Yellow points are detections missed by the spiking version (false negatives) and red points are false positive detections by the spiking algorithm. The SNNs were simulated with 250 time steps.

For SNNs that use temporal coding, especially the binning of spike times to time steps can become a severe constraint: When considering digital neuromorphic systems that have a global system tick for updating neurons or inserting spikes (such as Loihi, TrueNorth, or SpiNNaker), the total number of time steps will have a major impact on the accuracy of the time-coded spiking CFAR networks. To assess this limitation we implement the CFAR SNNs with different number of time steps where the input values are translated to discrete spike times. We compare the output of the spiking CFAR to the reference implementation and provide exemplary results: Figure 6A shows a challenging sample RD map from the CARRADA dataset due to the long extension of the main object and the slow degradation of the intensity until it becomes background. Figures 6B,C show the performance of the spiking OS- and CA-CFAR, respectively, when simulated for 250 time steps. We then count the number of true positives (TP: targets detected by both classical and spiking algorithms), false positives (FP: targets detected by spiking CFAR but not by conventional), and false negatives (FN: true targets not identified by spiking CFAR). The examples show many true positives, as well as several false negatives and few false positives. The detected bins by the classical algorithms differ slightly between OS and CA-CFAR due to the different approaches for noise level estimation. It further stands out that the spiking OS-CFAR has more false negatives than the spiking CA-CFAR but no false positive detections. Details on the chosen CFAR parameters are given in Supplementary Section 1.1.

For a statistical analysis we evaluated the spiking CFAR on 1,000 randomly selected RD maps from the CARRADA dataset and accumulated the counts of TP, FN and FP detections. Based on this we obtain the sensitivity and precision as performance indicators of the spiking variants:

(13) $$\begin{array}{r} \text{sensitivity}=\frac{TP}{TP+FN}, \end{array}$$

(14) $$\begin{array}{r} \text{precision}=\frac{TP}{TP+FP}. \end{array}$$

**Figure 7:**
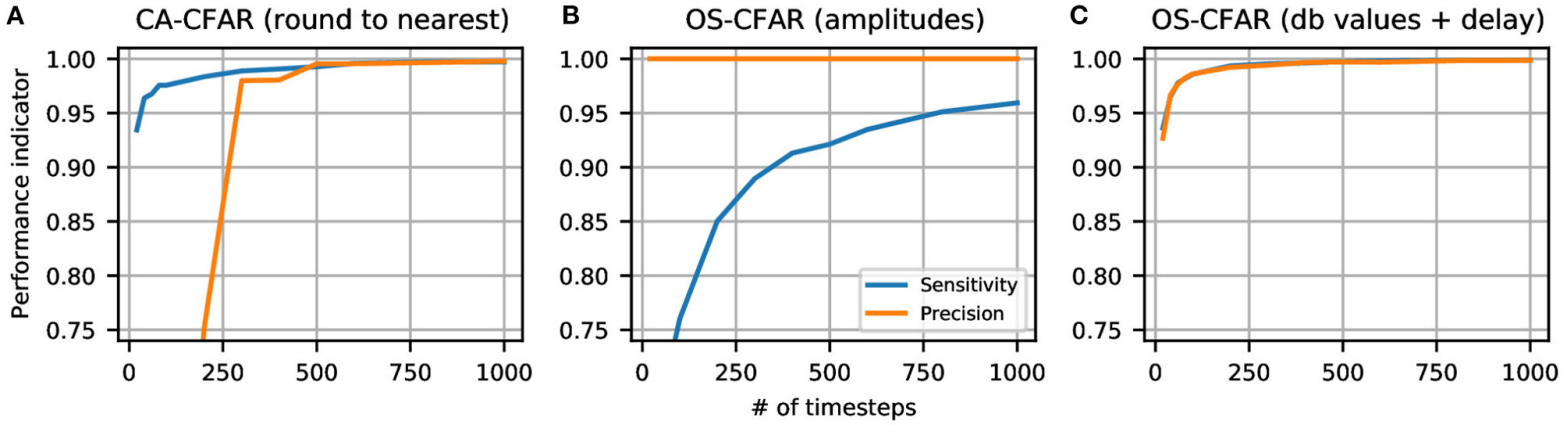
Evaluation of spiking CFAR regarding number of SNN time steps. **(A)** Spiking CA-CFAR with nearest rounding to discrete time steps. **(B)** Spiking OS-CFAR with range-Doppler map amplitudes as input. **(C)** Spiking OS-CFAR with dB values as input and a delay added to time steps of training cells. The evaluation was performed on 1000 range-Doppler maps from the CARRADA dataset.

For both metrics a value close or equal to 1 is desired. The metrics are evaluated depending on the number of SNN simulation time steps in Figure 7: the spiking CA-CFAR with nearest-rounding for binning spike times to simulation time steps shows a very high sensitivity even for less than 100 time steps, which means that only few CFAR detections are missed by the SNN. However, there are many false positive detections, so that the precision stays below 95% for less than 300 time steps. Only starting from 500 time steps both sensitivity and precision are above 99%, which we consider competitive to the original algorithm. We tried additional rounding schemes for the CA-CFAR, which are introduced and discussed in Supplementary Section 1.2.2, exhibiting a worse performance than the rounding to nearest presented here.

In contrast, the results of the spiking OS-CFAR in Figure 7B show a different dependency: There are no false positives at all (precision is always 1) while the sensitivity increases very slowly with the number of time steps and reaches 95% at around 800 time steps. The reason for this bad performance of the spiking OS-CFAR can be explained by looking at the distribution of input values: When using RD map amplitudes as inputs, most of the converted spike times are binned to only a few number of time steps which leads to missed detections by the SNN (see Supplementary Section 1.3.1 for details). Alternative rounding schemes are not considered here, as they do not affect the order statistic. Instead, we converted the RD map to a logarithmic scale prior to feeding the values to the network. Moreover, we added a small time delay to the neighbor cells in order to avoid false negatives when the center cell is slightly bigger than the *k*-th largest value. These modifications increased the sensitivity of the spiking network to around 99% when simulating it for 100 time steps (see Figure 7C). Further details and explanations are provided in Supplementary Section 1.3.

To sum up, we found that the performance of the algorithms can reach values close to 99% when using an adequate amount of time steps. The required time steps are lower for the spiking OS-CFAR, mostly thanks to the logarithmic re-scaling of the input range-Doppler map (which is not possible for the CA-CFAR). We note that, when the spiking CFAR algorithms are embedded into a full radar processing chain with subsequent classification and tracking, a lower performance with respect to the classical CFAR might be sufficient: One could co-optimize the parameters of the spiking CFAR and the classification algorithm to achieve a high overall performance, e.g., one could decrease the CFAR threshold factor to create more detections and let the classifier filter out the noise from actual radar targets.

Finally, we compare the computational effort of the spiking CFAR algorithms to the conventional approaches. For SNNs the effort mainly depends on the number of neuron updates and synaptic events. Both spiking CFAR networks perform as many neuron updates as time steps *N*_steps_ and process as many synaptic events as training cells *N*_train_. Additional effort is required for releasing input spikes at the predefined times, which, however, only needs to be done once per RD map bin if the CFAR is realized by one large SNN for the whole RD map. The classical CA-CFAR is dominated by *N*_train_ ADD operations. The OS-CFAR, which compares the *k*th largest element with the cell under test, can be efficiently implemented by *N*_train_ compare operations (a sorting of training cells is not required).

Table 1 evaluates the computational cost in terms of ADD and compare operations: The spiking OS-CFAR requires approximately *N*_steps_ more operations than the classic approach, and the spiking CA-CFAR needs 2*N*_steps_ more operations. Considering that in our example there are 176 training cells and 100 time steps (OS-CFAR) resp. 500 time steps (CA-CFAR), it is apparently not beneficial to realize the CFAR algorithms as spiking networks on conventional processors. Yet, a spiking CFAR network might be realized very efficiently on dedicated neuromorphic hardware, especially when the input is already provided as spikes, or when the spiking output is directly fed into subsequent SNNs. For the future, we suggest to directly compare the energy and latency of the spiking CFAR on neuromorphic hardware to the conventional CFAR on a suitable DSP.

**Table 1:** Comparison of computational cost of spiking and conventional CFAR algorithms.

**Algorithm**	**ADD**	**CMP**	**Total (this example)**
OS-CFAR	-	*N*_train_+1	177
OS-CFAR (spiking)	*N*_train_	*N*_steps_	276
CA-CFAR	*N*_train_	1	177
CA-CFAR (spiking)	*N*_train_+*N*_steps_	*N*_steps_	1176

*ADD: number of addition operations or synaptic events, CMP: number of compare operations. Total: total number of operations in the example network with N_train_= 176, N_steps_= 100 for OS-CFAR and N_steps_= 500 for CA-CFAR. The cost for releasing input spikes is discarded as being hard to quantify*.

### 4.2. Target Classification in Range-Doppler Maps

To evaluate the feasibility of spiking networks for object classification based on range-Doppler maps, the CARRADA dataset is used. As the conceived network is only for the purpose of object classification (and not object detection or localization), we prepare a sub-dataset that only contains fixed sized regions of interest for all labeled objects in the range-Doppler maps of the dataset. To include temporal dependencies the extracted regions are taken as small sequences of 8 frames. The generated sub-dataset contains 399 car, 208 bicycle, and 323 pedestrian sequences. Additionally, we consider the dataset of all single ROIs for training a 2D convolutional network as a baseline reference. The dataset preparation is elaborated in the Supplementary Section 2.1. We note that in a real-time scenario the ROIs need to be selected dynamically based on the location of detected objects.

**Figure 8:**
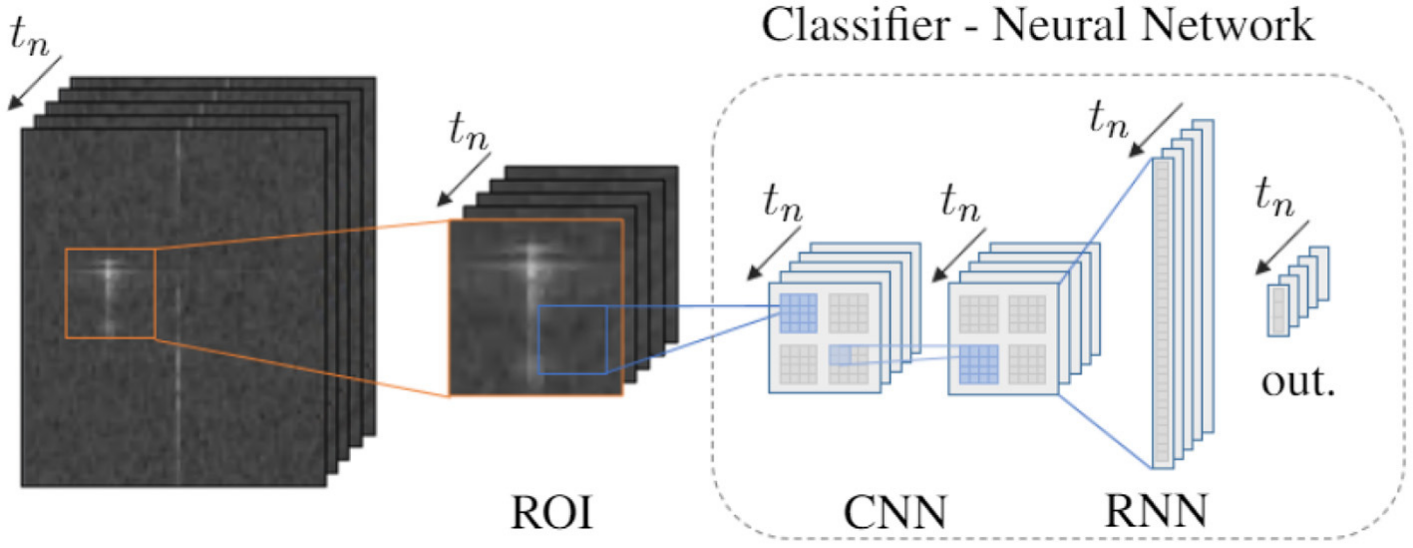
Approach and network architecture for radar object classification: From range-Doppler maps region of interests (ROIs) around detected objects are extracted and injected into a classifier neural network over discrete time steps *t*_*n*_. The network consists of two 2D convolutional layers, a recurrent layer and an output layer. Both ANN and SNN variants are compared, see main text for details.

Figure 8 visualizes the proposed network architecture for the classification of ROI sequences. Two variants of the neural network are considered: First, an ANN consisting of two convolutional layers, a recurrent layer with LSTM cells, and the output layer. Second, an SNN with two spiking convolutional layers, a recurrent layer with LIF neurons, and an output layer with non-spiking integrator neurons. Both networks have the same structure and layer sizes which are detailed in Supplementary Section 2.3. In both cases, the 2D convolutional layers extract spatial information from single frames while the recurrent layer combines the latter for spatio-temporal signal processing. The proposed SNN model resembles the spiking convolutional network for gesture classification from Safa et al. (2021b); yet it was developed independently and differs from it by having recurrent connections between the LIF neurons. Both the ANN and SNN in this work are trained on real-valued inputs and on event-encoded inputs. This allows to analyse the effect of converting the input data to spikes on the overall network performance. To generate the input spikes, the range-Doppler map values are compared to a specific reference value. With this scheme, on average 537 input spikes are used to encode the sequential ROIs including 8 discrete time steps. In case of the ANN the encoding is used to create a binary input map. In contrast, the first convolutional layer of the spiking network is also evaluated using real-numbered inputs in combination with a spiking activation function similar to some converted SNNs (Hunsberger and Eliasmith, 2016; Rueckauer et al., 2017). The network models are trained with BPTT, using surrogate gradients in case of the SNN. In addition to the recurrent networks, a 2D CNN is trained on single frames providing a baseline reference. Further details on the network architectures and training methods can be found in Supplementary Section 2.

Table 2 shows the results of the conducted experiments: The best accuracy of 94.7% is achieved by the ANN with convolutional and LSTM layers applied on ROI sequences, significantly better than the CNN on single frames (90.5%) and the pure SNN with spike input (90%). When the recurrent ANN is trained with binary inputs, the accuracy drops down to 86.3%. In contrast, when the SNN processes the first convolutional layer with real-valued inputs, the accuracy is increased to 92.6% getting closer to the ANN. The table also provides the number of parameters and the number of operations [spikes, synaptic events, and multiply-accumulate (MAC) operations] per network model as a measure of the network complexity and computational cost. The SNN with spike input clearly shows the best compromise between number of operations and achieved accuracy. Also, the SNN with real-numbered inputs still requires less than 20% of operations of the best performing ANN.

**Table 2:** Radar object classification results.

**Architecture**	**Parameter**	**Acc**.	**Avg. Spikes**	**Avg. Syn. Events**	**MAC ops**
CNN (single frame)	11 593	90.5%	-	-	94 398
ANN (real-valued input)	46 256	94.7%	-	-	1 031 680
ANN (spike input)	46 256	86.3%	-	-	1 031 680
SNN (real-valued input)	12 303	92.6%	145	1990	186 624
SNN (spike input)	12 303	90.0%	642	3944	-

Note that, in contrast to the spiking CFAR evaluation, we do not consider specific limitations of neuromorphic hardware like the number of time steps, which is 8 in this experiment for all recurrent networks. Yet, the results show that the binary spike encoding decreases the accuracy for both the ANN and SNN. We further remark that the dataset is rather small and we expect that the test accuracy can be improved for all models by increasing the training data and a thorough hyperparameter optimization. Nonetheless, this example demonstrates the general feasibility of SNNs for efficient object classification with automotive radars.

## 5. Discussion

### 5.1. Summary, Related Work and Limitations

In this article, we reviewed the state-of-the-art digital signal processing steps for automotive radars and discussed for each step various SNN approaches as replacements (Section 3). To the best of our knowledge, such comprehensive analysis of concepts for radar processing with SNNs has not been done before. Yet, we consider this collection of approaches preliminary, and we are sure that more and enhanced approaches will be adopted or developed in the future. Furthermore, for two processing steps we have provided concrete SNN examples and compared them to classical approaches: For the CFAR object detection we developed two temporally coded SNNs and analyzed their accuracy depending on time steps. Starting from 100 time steps, the spiking version is competitive with the reference approach. For object classification, we trained a deep recurrent SNN with BPTT and surrogate gradients on ROI sequences of range-Doppler maps from the CARRADA dataset. The accuracy of the SNN with real-valued inputs of 92.6% is close to the 94.7% achieved by a reference ANN while requiring only 18% of the operations. Instead, the pure SNN with spike input achieves 90.0% with less than 0.5% of the operation of the ANN. Further improvement is expected by increasing the size of the dataset and performing a systematic hyperparameter search. Regarding related work in the context of FMCW radar, so far, SNNs have only been used for gesture recognition, cf. Section 3.4. Very recently, Stuijt et al. (2021) have demonstrated radar gesture recognition using an ultra-low-power SNN chip and a 8 GHz FMCW radar. They turn the micro-Doppler map into a small binary image and classify it with a rate-based feed-forward SNN on the chip. In López-Randulfe et al. (2022), the time-coded spiking Fourier transform introduced in Section 3.1.2 was implemented and validated on Loihi to compute the range and Doppler-FFT on recorded radar data. Compared to dedicated hardware FFT accelerators, the neuromorphic solution lags behind by one to three orders of magnitudes in terms of energy and latency. Brown et al. (2021) have developed an SNN hardware accelerator for compressed sensing with pulse-Doppler radars. A spiking locally competitive algorithm (LCA) solves the sparse optimization to achieve highly accurate and efficient target and velocity estimation. This compressed sensing approach is not directly applicable to the FMCW automotive radar processing chain discussed in this article. Further, Barnell et al. (2020) use spiking DNNs on Loihi for classification of synthetic aperture radar images. While this demonstrates the efficiency of neuromorphic hardware for image classification, new network models will have to be developed for automotive FMCW radar data.

The SNN concepts presented in this work apply to *single steps* of the radar processing chain. How to combine several SNNs or how to build a radar processing chain completely with spiking neurons was not the objective of this paper and remains an open research subject.

### 5.2. Toward Neuromorphic Radar Sensors

Whether or not spiking neural networks can outperform conventional radar processing depends on how efficiently they can be realized in neuromorphic hardware. In the following, we summarize the requirements of a neuromorphic application-specific integrated circuit (ASIC) to process the radar data in real time. For this, we assume that a neuromorphic processor replaces or complements a DSP (cf. Figure 2A) and receives raw ADC data or preprocessed data that has to be converted to spikes on the chip. Our analysis includes the required memory for buffering input data, the required input bandwidth, the number of neurons and synapses, and the processing speed of those neuromorphic components. As a radar sensor setup we take the one from the CARRADA dataset with 2 transmitters and 4 receivers (cf. Table 3), yet we note that the requirements for high-resolution radars will strongly increase. Reviewing the radar processing steps from Figure 3, the hardware requirements vary significantly for each processing step, e.g., the amount of input data per frame that needs to be processed varies a lot, as shown in Table 3. Especially processing the full raw data or high-resolution range-angle maps requires more than 100 kB of memory for buffering the input. This amount does not pose a problem for typical embedded micro-processors, yet it might become challenging for high-resolution radars with more than 10 times as much data or when fed into edge neuromorphic processors. Similarly, for the communication between a radar sensor and neuromophic hardware at least a bandwidth of 10–100 MBit/s is needed.

**Table 3:** Requirements for a neuromorphic ASIC for radar processing.

**Frame parameter**	**Value**				
*N*_TX_	2				
*N*_RX_	4				
*N*_chirps_	64				
*N*_samples_ (complex)	256				
Frames per second	10				
**Input type**		**Memory [kB]**	**Bandwidth [MBit/s]**		
Raw data cube (256x64x4 á 2x16b)		197	15.7		
Range-Doppler map (256x64 á 16b)		32.8	2.62		
Range-angle map (256x256 á 16b)		131	10.5		
**Processing step**	**Inputs**	**Neurons**	**Synapses**	**Time steps/ frame**	**Repetitions/frame**
Range-FFT (López-Randulfe et al., 2022)	512	4608	37 888	550	256
OS-CFAR on RD map (this work)	16 384	16 384	2 899 968	100	1
Object classification (this work, pure SNN)	520	3747	311 328	1	1-20

At the bottom of Table 3, we review SNN requirements for some of the radar processing steps: The range S-FT with time coding from López-Randulfe et al. (2022) can be realized with sparse connectivity and one spike per synaptic connection for 550 time steps. While the S-FT network itself is rather small, the challenge is to run the model 256 times (64 chirps × 4 receivers) per frame on a neuromorphic processor (e.g., within 20 ms assuming that 20% of the 100 ms frame time are budgeted for the range-FFT). This seems possible, according to the results obtained in López-Randulfe et al. (2022), where a 1024-point spiking FFT can be calculated every 105 μs on the Loihi neuromorphic chip. For the *spiking OS-CFAR* from Section 4.1, a network of 16 k input and output neurons with nearly 3 million synapses is required to process an entire range-Doppler map. Compared to the range-FFT, this SNN is run only once per frame and thus has lower neuromorphic compute demands. Finally, the SNN-based radar object classification (Section 4.2) has the least requirements for implementation on neuromorphic hardware as the network is smaller and there is only one time step per frame (cf. Supplementary Section 2.3). Note, however, that the network needs to be newly instantiated for each detected object and we expect in the order of up to 20 radar objects in simple street scenes.

Looking at the neuromorphic requirements for the different automotive radar processing steps, we expect that SNN-based object classification has the highest potential for energy-efficient realization in neuromorphic hardware. SNN-based object tracking should also be evaluated further in the future. For the earlier processing steps like the FT and CFAR object detection, further work shall determine if neuromorphic hardware tailored at these operations can implement these operations more efficiently than digital signal processors and close the current gap in terms of energy and time performance (López-Randulfe et al., 2022). At the system level, one could alternatively combine a DSP with a neuromorphic processor to achieve maximum efficiency. When split onto different chips, the data bandwidth requirements from Table 3 need to be fulfilled. An even more radical approach for radar processing with neuromorphic hardware is to use analog spiking neurons in hardware with the radar IF signal as input. Resonate-and-fire neurons are the perfect candidates for that, but this might be limited to radar systems that don't need phase information, e.g., using a single transmitter and receiver. Yet, further research may clarify whether a full SNN pipeline on dedicated neuromorphic hardware can outperform classical DSP or hybrid DSP/ANN approaches.

### 5.3. Toward Neuromorphic Automated Driving

As motivated in the introduction, the use of neuromorphic hardware has a high potential to significantly reduce the energy demands for highly-automated driving. Besides radar signals, also camera and LIDAR data need to be processed in order to get a complete understanding of the automotive scene. For image processing there already exist first attempts to solve complex tasks with SNNs, e.g., for object detection (Kim et al., 2020) or semantic segmentation (Kim et al., 2021). Also recently, Viale et al. (2021) realized an SNN on Loihi for car detection using a dynamic vision sensor. Using LIDAR data, which is naturally sparse and thus predestined for SNNs, Zhou et al. (2020) showed a spiking convolutional network for real-time 3D object detection. Shalumov et al. (2021) use LIDAR data for SNN-based collision avoidance with a control network based on the neural engineering framework. All these examples show that SNN-based sensor processing for autonomous driving is a trending topic. Besides the development of SNNs and their implementation on neuromorphic hardware, also the combined processing, i.e., sensor fusion using SNNs, will become an important topic.

When it comes to AI-based autonomous driving, ensuring functional safety of both software and hardware is a critical issue. The principles that are currently developed to support machine learning models (Henriksson et al., 2018; Mohseni et al., 2019) will also apply to SNNs. Similarly, neuromorphic hardware will have to fulfill the same standards as any automotive electronic system: adhere to temperature ranges, be resistant to vibrations, be deterministic and redundant, or contain self-monitoring. For that reason, only digital neuromorphic systems are candidates for integration in cars, while the use of analog or mixed-signal neuromorphic hardware seems out of scope at the moment due to their intrinsic variability. Hence, we suggest to focus on advanced digital systems such as SpiNNaker2 (Yan et al., 2021) or Loihi2 (Orchard et al., 2021) to further explore neuromorphic hardware for automotive radar processing and automated driving in general.

## Data Availability Statement

Publicly available datasets were analyzed in this study. The CARRADA dataset used in this study can be found at: https://github.com/valeoai/carrada_dataset. The source code for running the experiments in this article is available at https://gitlab.com/ki-asic/carrada-snn.

## Author Contributions

BV, FK, JL-R, CL, RD, HG, DS, NR, DA, FM, JH, and MA wrote the initial manuscript and contributed to the analysis of SNN approaches for automotive radar processing. BV developed the spiking CA-CFAR algorithm. BV and JL-R performed the spiking CFAR experiments and developed the improved spiking OS-CFAR variant with logarithmic input. FK, CL, and BV developed the SNN for radar object classification and performed the comparison experiments. BV derived the requirements for neuromorphic radar sensors. BV, JL-R, and DS drafted the discussion. CG, AK, and CM provided technical and scientific advice and organized funding. All authors contributed to the article and approved the submitted version.

## Funding

This work was funded by the German Federal Ministry of Education and Research (BMBF) within the KI-ASIC project (16ES0995, 16ES0996, 16ES0993, 16ES0992K, and 16ES0994). This work was partially funded by the German Research Foundation (DFG, Deutsche Forschungsgemeinschaft) as part of Germany's Excellence Strategy – EXC 2050/1 – Project ID 390696704 – Cluster of Excellence Centre for Tactile Internet with Human-in-the-Loop (CeTI) of Technische Universität Dresden. The authors also acknowledge the financial support by the Federal Ministry of Education and Research of Germany in the programme of Souverän. Digital. Vernetzt. Joint project 6G-life, Project Identification Number: 16KISK001K.

## Conflict of Interest

FK and DS were employed by Infineon Technologies Dresden GmbH & Co., KG. DA, JH, MA, and CG were employed by Infineon Technologies AG. FM was employed by BMW Group. The remaining authors declare that the research was conducted in the absence of any commercial or financial relationships that could be construed as a potential conflict of interest.

## Publisher's Note

All claims expressed in this article are solely those of the authors and do not necessarily represent those of their affiliated organizations, or those of the publisher, the editors and the reviewers. Any product that may be evaluated in this article, or claim that may be made by its manufacturer, is not guaranteed or endorsed by the publisher.

## References

[B1] Aeberhard M. Rauch S. Bahram M. Tanzmeister G. Thomas J. Pilat Y.. (2015). Experience, results and lessons learned from automated driving on germany's highways. IEEE Intell. Transp. Syst. Mag. 7, 42–57. 10.1109/MITS.2014.2360306

[B2] Angelov A. Robertson A. Murray-Smith R. Fioranelli F. (2018). Practical classification of different moving targets using automotive radar and deep neural networks. Sonar Navig. IET Radar 12, 1082–1089. 10.1049/iet-rsn.2018.0103

[B3] Arkind N. Baron A. Stettiner Y. (2020). Compact radar switch/MIMO array antenna with high azimuth and elevation angular resolution. U.S. Patent App. 16/480,030.

[B4] Arsalan M. Chmurski M. Santra A. El-Masry M. Weigel R. Issakov V. (2021). Resource efficient gesture sensing based on fmcw radar using spiking neural networks, in 2021 IEEE MTT-S International Microwave Symposium.

[B5] Auge D. Hille J. Kreutz F. Mueller E. Knoll A. (2021a). End-to-end spiking neural network for speech recognition using resonating input neurons. In International Conference on Artificial Neural Networks, pages 245–256. Springer.

[B6] Auge D. Hille J. Mueller E. Knoll A. (2021b). A survey of encoding techniques for signal processing in spiking neural networks. Neural Process. Lett., 1–18.

[B7] Auge D. Mueller E. (2020). Resonate-and-Fire Neurons As Frequency Selective Input Encoders for Spiking Neural Networks. Technical Report TUM-I2083, TU Munich, Munich.

[B8] Badrinarayanan V. Kendall A. Cipolla R. (2015). SegNet: a deep convolutional encoder-decoder architecture for image segmentation. Comput. Res. Repository (CoRR), abs/1511.00561. 10.1109/TPAMI.2016.2644615 28060704

[B9] Banerjee D. Rani S. George A. M. Chowdhury A. Dey S. Mukherjee A.. (2020). Application of spiking neural networks for action recognition from radar data, in 2020 International Joint Conference on Neural Networks (IJCNN) (IEEE), 1–10.

[B10] Barnell M. Raymond C. Wilson M. Isereau D. Cicotta C. (2020). Target classification in synthetic aperture radar and optical imagery using loihi neuromorphic hardware, in 2020 IEEE High Performance Extreme Computing Conference (HPEC), 1–6.

[B11] Bartsch A. Fitzek F. Rasshofer R. (2012). Pedestrian recognition using automotive radar sensors. Adv. Radio Sci. ARS, 10, 45–55. 10.5194/ars-10-45-2012

[B12] Bellec G. Scherr F. Subramoney A. Hajek E. Salaj D. Legenstein R. Maass W. (2020). A solution to the learning dilemma for recurrent networks of spiking neurons. Nat. Commun. 11, 1–15. 32681001 10.1038/s41467-020-17236-y PMC7367848

[B13] Bi G.-Q. Poo M.-M. (1998). Synaptic modifications in cultured hippocampal neurons: dependence on spike timing, synaptic strength, and postsynaptic cell type. J. Neurosci. 18, 10464–10472. 9852584 10.1523/JNEUROSCI.18-24-10464.1998 PMC6793365

[B14] Bilik I. Villeval S. Brodeski D. Ringel H. Longman O. Goswami P.. (2018). Automotive multi-mode cascaded radar data processing embedded system, in 2018 IEEE Radar Conference (RadarConf18), 0372–0376.

[B15] Bohte S. M. Poutre H. L. Kok J. N. (2002). Unsupervised clustering with spiking neurons by sparse temporal coding and multilayer RBF networks. IEEE Trans. Neural Netw. 13, 426–435. 10.1109/72.991428 18244443

[B16] Brandli C. Berner R. Yang M. Liu S.-C. Delbruck T. (2014). A 240 × 180 130 db 3 μs latency global shutter spatiotemporal vision sensor. IEEE J. Solid-State Circuits 49, 2333–2341. 10.1109/JSSC.2014.2342715

[B17] Brown P. L. O'Shaughnessy M. Rozell C. Romberg J. Flynn M. (2021). A 17.8-ms/s compressed sensing radar accelerator using a spiking neural network. IEEE J. Solid-State Circuits 56, 834–843. 10.1109/JSSC.2020.3025864

[B18] Caesar H. Bankiti V. Lang A. H. Vora S. Liong V. E. Xu Q.. (2019). nuScenes: a multimodal dataset for autonomous driving. arXiv:1903.11027 [cs, stat].

[B19] Capobianco S. Facheris L. Cuccoli F. Marinai S. (2018). Vehicle classification based on convolutional networks applied to FMCW radar signals, in Traffic Mining Applied to Police Activities, Advances in Intelligent Systems and Computing, eds Leuzzi F. Ferilli S. (Cham: Springer International Publishing), 115–128.

[B20] Carr C. Konishi M. (1990). A circuit for detection of interaural time differences in the brain stem of the barn owl. J. Neurosci. 10, 3227–3246. 2213141 10.1523/JNEUROSCI.10-10-03227.1990 PMC6570189

[B21] Chan V. Liu S.-C. van Schaik A. (2007). Aer ear: A matched silicon cochlea pair with address event representation interface. IEEE Trans. Circuits Syst. I Reg. Papers 54, 48–59.

[B22] Ciresan D. C. Meier U. Masci J. Schmidhuber J. (2012). Multi-column deep neural network for traffic sign classification. Neural Netw. 32, 333–338. 10.1016/j.neunet.2012.02.023 22386783

[B24] Cooley J. W. Tukey J. W. (1965). An algorithm for the machine calculation of complex fourier series. Math. Comput. 19, 297–301.

[B25] Davies M. N. S. Lin T. H. Chinya G. Cao Y. Choday S. H. Dimou G.. (2018). Loihi: a neuromorphic manycore processor with on-chip learning. IEEE Micro 38, 82–99. 10.1109/MM.2018.112130359

[B26] Davies M. Wild A. Orchard G. Sandamirskaya Y. Guerra G. A. F. Joshi P.. (2021). Advancing neuromorphic computing with loihi: a survey of results and outlook. Proc. IEEE 109, 911–934. 10.1109/JPROC.2021.3067593

[B27] Deo N. Trivedi M. M. (2018). Multi-modal trajectory prediction of surrounding vehicles with maneuver based lstms, in 2018 IEEE Intelligent Vehicles Symposium (IV) (Changshu: IEEE), 1179–1184.

[B28] Desimone R. Duncan J. (1995). Neural mechanisms of selective visual attention. Annu. Rev. Neurosci. 18, 193–222. 7605061 10.1146/annurev.ne.18.030195.001205

[B29] Diamond A. Schmuker M. Nowotny T. (2019). An unsupervised neuromorphic clustering algorithm. Biol. Cybern. 113, 423–437. 10.1007/s00422-019-00797-7 30944983 PMC6658584

[B30] Ebrahimpour M. K. Li J. Yu Y.-Y. Reesee J. Moghtaderi A. Yang M.-H. Noelle D. C. (2019). Ventral-dorsal neural networks: object detection via selective attention, in 2019 IEEE Winter Conference on Applications of Computer Vision (WACV) (Waikoloa, HI), 986–994.

[B31] Eliasmith C. Anderson C. H. (2003). Neural Engineering: Computation, Representation, and Dynamics in Neurobiological Systems. Cambridge, MA: MIT Press

[B32] Ester M. Kriegel H.-P. Sander J. Xu X.. (1996). A density-based algorithm for discovering clusters a density-based algorithm for discovering clusters in large spatial databases with noise, in Proceedings of the Second International Conference on Knowledge Discovery and Data Mining KDD'96 (AAAI Press) 226–231.

[B33] Etienne A. S. Jeffery K. J. (2004). Path integration in mammals. Hippocampus 14, 180–192. 10.1002/hipo.10173 15098724

[B34] Frady E. P. Orchard G. Florey D. Imam N. Liu R. Mishra J.. (2020). Neuromorphic Nearest Neighbor Search Using Intel's Pohoiki springs, in Proceedings of the Neuro-Inspired Computational Elements Workshop NICE '20 (New York, NY: Association for Computing Machinery), 1–10.

[B35] Frémaux N. Gerstner W. (2016). Neuromodulated spike-timing-dependent plasticity, and theory of three-factor learning rules. Front. Neural Circuits 9, 85. 10.3389/fncir.2015.00085 26834568 PMC4717313

[B36] Furber S. (2016). Large-scale neuromorphic computing systems. J. Neural Eng. 13, 051001. 10.1088/1741-2560/13/5/051001 27529195

[B37] Furber S. B. Galluppi F. Temple S. Plana L. A. (2014). The spinnaker project. Proc. IEEE 102, 652–665. 10.1109/JPROC.2014.2304638

[B38] Gamba J. (2020). Radar Signal Processing for Autonomous Driving. Singapore: Springer.

[B39] Gawron J. H. Keoleian G. A. De Kleine R. D. Wallington T. J. Kim H. C. (2018). Life cycle assessment of connected and automated vehicles: sensing and computing subsystem and vehicle level effects. Environ. Sci. Technol. 52, 3249–3256. 10.1021/acs.est.7b04576 29446302

[B40] Geiger A. Lenz P. Stiller C. Urtasun R. (2013). Vision meets robotics: the kitti dataset. Int. J. Robot. Res. (IJRR) 32, 1231–1237. 10.1177/0278364913491297

[B41] Gentilho E. Scalassara P. R. Abrão T. (2019). Direction-of-arrival estimation methods: a performance-complexity tradeoff perspective. J. Signal Process. Syst. 92, 239–256. 10.1007/s11265-019-01467-4

[B42] Ginsburg B. P. Subburaj K. Samala S. Ramasubramanian K. Singh J. Bhatara S.. (2018). A multimode 76-to-81ghz automotive radar transceiver with autonomous monitoring, in 2018 IEEE International Solid - State Circuits Conference - (ISSCC) (San Francisco, CA), 158–160.

[B43] Göltz J. Kriener L. Baumbach A. Billaudelle S. Breitwieser O. Cramer B.. (2021). Fast and energy-efficient neuromorphic deep learning with first-spike times. Nat. Mach. Intell. 3, 823–835. 10.1038/s42256-021-00388-x

[B44] Gonzalez H. A. Liu C. Vogginger B. Kumaraveeran P. Mayr C. G. (2021). Doppler disambiguation in mimo fmcw radars with binary phase modulation. IET Radar Sonar Navig. 15, 884–901. 10.1049/rsn2.12063

[B45] Gordon N. J. Salmond D. J. Smith A. F. (1993). Novel approach to nonlinear/non-Gaussian Bayesian state estimation, in IEE Proceedings F (Radar and Signal Processing), Vol. 140 (Toulon: IET), 107–113.

[B46] Henriksson J. Borg M. Englund C. (2018). Automotive safety and machine learning: initial results from a study on how to adapt the iso 26262 safety standard, in 2018 IEEE/ACM 1st International Workshop on Software Engineering for AI in Autonomous Systems (SEFAIAS) (Gothenburg: IEEE), 47–49.

[B47] Heuel S. Rohling H. (2011). Two-stage pedestrian classification in automotive radar systems, in 2011 12th International Radar Symposium (IRS) (Leipzig), 477–484.

[B48] Heuel S. Rohling H. (2012). Pedestrian classification in automotive radar systems, in 2012 13th International Radar Symposium (Warsaw), 39–44.

[B23] Hinneburg A. Keim D. A.. (1998). An efficient approach to clustering in large multimedia databases with noise, in KDD, Vol. 98, 58–65.

[B49] Hopfield J. J. (1995). Pattern recognition computation using action potential timing for stimulus representation. Nature 376, 33–36. 7596429 10.1038/376033a0

[B50] Hunsberger E. Eliasmith C. (2016). Training spiking deep networks for neuromorphic hardware. arXiv preprint arXiv:1611.05141.

[B51] Ikram M. Z. Ali M. (2013). 3-d object tracking in millimeter-wave radar for advanced driver assistance systems, in 2013 IEEE Global Conference on Signal and Information Processing (Austin, TX: IEEE), 723–726.

[B52] Indiveri G. Linares-Barranco B. Hamilton T. van Schaik A. Etienne-Cummings R. Delbruck T.. (2011). Neuromorphic silicon neuron circuits. Front. Neurosci. 5, 73. 10.3389/fnins.2011.00073 21747754 PMC3130465

[B53] Izhikevich E. M. (2001). Resonate-and-fire neurons. Neural Netw. 14, 883–894. 11665779 10.1016/s0893-6080(01)00078-8

[B54] Jiménez-Fernández A. Cerezuela-Escudero E. Miró-Amarante L. Domínguez-Morales M. J. de Asís Gómez-Rodríguez F. Linares-Barranco A.. (2016). A binaural neuromorphic auditory sensor for FPGA: a spike signal processing approach. IEEE Trans. Neural Netw. Learn. Syst. 28, 804–818. 10.1109/TNNLS.2016.2583223 27479979

[B55] Kalman R. E. (1960). A new approach to linear filtering and prediction problems. Trans. ASME J. Basic Eng. 82, 35–45. 30253628

[B56] Khalid F. B. Nugraha D. T. Roger A. Ygnace R. Bichl M. (2018). Distributed signal processing of high-resolution FMCW MIMO radar for automotive applications, in 2018 15th European Radar Conference (EuRAD) (Madrid), 513–516.

[B57] Kim S. Lee S. Doo S. Shim B. (2018). Moving Target classification in automotive radar systems using convolutional recurrent neural networks, in 2018 26th European Signal Processing Conference (EUSIPCO) (Rome), 1482–1486.

[B58] Kim S. Park S. Na B. Yoon S. (2020). Spiking-yolo: spiking neural network for energy-efficient object detection, in Proceedings of the AAAI Conference on Artificial Intelligence, Vol. 34, 11270–11277.

[B59] Kim Y. Chough J. Panda P. (2021). Beyond classification: directly training spiking neural networks for semantic segmentation. arXiv preprint arXiv:2110.07742.

[B60] Kim Y. Moon T. (2016). Human detection and activity classification based on micro-doppler signatures using deep convolutional neural networks. IEEE Geosci. Rem. Sens. Lett. 13, 8–12. 10.1109/LGRS.2015.2491329

[B61] Klarenbeek G. Harmanny R. I. A. Cifola L. (2017). Multi-target human gait classification using LSTM recurrent neural networks applied to micro-Doppler, in 2017 European Radar Conference (EURAD) (Nuremberg), 167–170.

[B62] Kreutz F. Gerhards P. Vogginger B. Knobloch K. Mayr C. G. (2021). Applied spiking neural networks for radar-based gesture recognition, in 2021 7th International Conference on Event-Based Control, Communication, and Signal Processing (EBCCSP) (Kraków), 1–4.

[B63] Lee S. Yoon Y.-J. Lee J.-E. Kim S.-C. (2017). Human–vehicle classification using feature-based SVM in 77-GHz automotive FMCW radar. Sonar Navig. IET Radar 11, 1589–1596. 10.1049/iet-rsn.2017.0126

[B64] Lichtsteiner P. Posch C. Delbruck T. (2008). A 128x128 120 db 15 μs latency asynchronous temporal contrast vision sensor. IEEE J. Solid-State Circuits 43, 566–576. 10.1109/JSSC.2007.914337

[B65] Lin P. Chang S. Wang H. Huang Q. He J. (2019). SpikeCD: A parameter-insensitive spiking neural network with clustering degeneracy strategy. Neural Comput. Appl. 31, 3933–3945. 10.1007/s00521-017-3336-6

[B66] Liu S.-C. van Schaik A. Minch B. A. Delbruck T. (2014). Asynchronous Binaural Spatial Audition Sensor With 2 x 64 x 4 Channel Output. IEEE Trans. Biomed. Circuits Syst. 8, 453–464. 10.1109/tbcas.2013.2281834 24216772

[B67] López-Randulfe J. Duswald T. Bing Z. Knoll A. (2021). Spiking neural network for fourier transform and object detection for automotive radar. Front. Neurorobot. 15, 688344. 10.3389/fnbot.2021.688344 34163347 PMC8216499

[B68] López-Randulfe J. Reeb N. Karimi N. Liu C. Gonzalez H. A. Dietrich R.. (2022). Time-coded spiking fourier transform in neuromorphic hardware. arXiv [Preprint].arXiv: 2202.12650. 10.48550/arXiv.2202.12650

[B69] Maass W. (1997). Networks of spiking neurons: the third generation of neural network models. Neural Netw. 10, 1659–1671.

[B70] Maass W. Natschläger T. Markram H. (2002). Real-time computing without stable states: a new framework for neural computation based on perturbations. Neural Comput. 14, 2531–2560. 10.1162/089976602760407955 12433288

[B71] Marr B. Degnan B. Hasler P. Anderson D. (2013). Scaling energy per operation via an asynchronous pipeline. IEEE Trans. Very Large Scale Integr. (VLSI) Syst. 21, 147–151. 10.1109/TVLSI.2011.2178126

[B72] Masquelier T. Guyonneau R. Thorpe S. J. (2008). Spike timing dependent plasticity finds the start of repeating patterns in continuous spike trains. PLoS ONE 3, e1377. 10.1371/journal.pone.0001377 18167538 PMC2147052

[B73] Mayr C. Hoeppner S. Furber S. (2019). Spinnaker 2: a 10 million core processor system for brain simulation and machine learning. arXiv[Preprint]. arXiv: 1911.02385. 10.48550/arXiv.1911.02385

[B74] Mead C. (1990). Neuromorphic electronic systems. Proc. IEEE 78, 1629–1636.

[B75] Merolla P. A. Arthur J. V. Alvarez-Icaza R. Cassidy A. S. Sawada J. Akopyan F.. (2014). A million spiking-neuron integrated circuit with a scalable communication network and interface. Science 345, 668–673. 10.1126/science.1254642 25104385

[B76] Meyer M. Kuschk G. (2019a). Automotive radar dataset for deep learning based 3D object detection, in 2019 16th European Radar Conference (EuRAD) (Paris), 129–132.

[B77] Meyer M. Kuschk G. (2019b). Deep learning based 3D object detection for automotive radar and camera, in 2019 16th European Radar Conference (EuRAD) (Paris), 133–136.

[B78] Milford M. Wyeth G. Prasser D. (2004). RatSLAM: a hippocampal model for simultaneous localization and mapping, in IEEE International Conference on Robotics and Automation, 2004. Proceedings. ICRA '04. 2004, Vol. 1 (New Orleans, LA), 403–408.

[B79] Mohseni S. Pitale M. Singh V. Wang Z. (2019). Practical solutions for machine learning safety in autonomous vehicles. arXiv preprint arXiv:1912.09630.

[B80] Mostafa H. (2017). Supervised learning based on temporal coding in spiking neural networks. IEEE Trans. Neural Netw. Learn. Syst. 29, 3227–3235. 10.1109/TNNLS.2017.2726060 28783639

[B81] Natschläger T. Ruf B. (1998). Spatial and temporal pattern analysis via spiking neurons. Netw. Computat. Neural Syst. 9, 319–332. 9861993

[B82] Neftci E. O. Mostafa H. Zenke F. (2019). Surrogate gradient learning in spiking neural networks: bringing the power of gradient-based optimization to spiking neural networks. IEEE Signal Process. Mag. 36, 51–63. 10.1109/MSP.2019.2931595

[B83] Och A. Pfeffer C. Schrattenecker J. Schuster S. Weigel R. (2018). A scalable 77 ghz massive mimo fmcw radar by cascading fully-integrated transceivers, in 2018 Asia-Pacific Microwave Conference (APMC) (Kyoto: IEEE), 1235–1237.

[B84] Orchard G. Frady E. P. Rubin D. B. D. Sanborn S. Shrestha S. B. Sommer F. T.. (2021). Efficient neuromorphic signal processing with loihi 2, in 2021 IEEE Workshop on Signal Processing Systems (SiPS) (Coimbra: IEEE), 254–259.

[B85] Ouaknine A. Newson A. Rebut J. Tupin F. Pérez P. (2020). Carrada dataset: Camera and automotive radar with range-angle-doppler annotations, in 2020 25th International Conference on Pattern Recognition, 5068–5075. 10.1109/ICPR48806.2021.9413181

[B86] Patel K. Rambach K. Visentin T. Rusev D. Pfeiffer M. Yang B. (2019). Deep learning-based object classification on automotive radar spectra, in 2019 IEEE Radar Conference (RadarConf) (Boston, MA: IEEE), 1–6.

[B87] Patole S. M. Torlak M. Wang D. Ali M. (2017). Automotive radars: a review of signal processing techniques. IEEE Signal Process. Mag. 34, 22–35. 10.1109/MSP.2016.2628914

[B88] Pérez R. Schubert F. Rasshofer R. Biebl E. (2019). Deep learning radar object detection and classification for urban automotive scenarios, in 2019 Kleinheubach Conference (Miltenberg: IEEE), 1–4.

[B89] Pfeil T. Scherzer A.-C. Schemmel J. Meier K. (2013). Neuromorphic learning towards nano second precision, in The 2013 International Joint Conference on Neural Networks (IJCNN) (Dallas, TX: IEEE), 1–5.

[B90] Qiao N. Mostafa H. Corradi F. Osswald M. Stefanini F. Sumislawska D.. (2015). A reconfigurable on-line learning spiking neuromorphic processor comprising 256 neurons and 128k synapses. Front. Neurosci. 9, 141. 10.3389/fnins.2015.00141 25972778 PMC4413675

[B91] Rao S. Subburaj K. Wang D. Ahmad A. (2020). Methods and Apparatus for Velocity Detection in MIMO Radar Including Velocity Ambiguity Resolution. U.S. Patent 10,627,483.

[B92] Rasmussen D. (2019). Nengodl: combining deep learning and neuromorphic modelling methods. Neuroinformatics 17, 611–628. 10.1007/s12021-019-09424-z 30972529

[B93] Redish A. D. Touretzky D. S. (1997). Cognitive maps beyond the hippocampus. Hippocampus 7, 15–35. 9138665 10.1002/(SICI)1098-1063(1997)7:1<15::AID-HIPO3>3.0.CO;2-6

[B94] Redmon J. Divvala S. Girshick R. Farhadi A. (2016). “You only look once: unified, real-time object detection,”' in Proceedings of the IEEE Conference on Computer Vision and Pattern Recognition (Las Vegas, NV), 779–788.

[B95] Rohling H. (1983). Radar CFAR thresholding in clutter and multiple target situations. IEEE Trans. Aerosp. Electron. Syst. AES-19, 608–621.

[B96] Roos F. Bechter J. Knill C. Schweizer B. Waldschmidt C. (2019). Radar sensors for autonomous driving: modulation schemes and interference mitigation. IEEE Microw. Mag. 20, 58–72. 10.1109/MMM.2019.2922120

[B97] Roy K. Jaiswal A. Panda P. (2019). Towards spike-based machine intelligence with neuromorphic computing. Nature 575, 607–617. 10.1038/s41586-019-1677-2 31776490

[B98] Roy R. Kailath T. (1989). Esprit-estimation of signal parameters via rotational invariance techniques. IEEE Trans. Acoust. Speech Signal Process. 37, 984–995. 26571506

[B99] Rueckauer B. Lungu I.-A. Hu Y. Pfeiffer M. Liu S.-C. (2017). Conversion of continuous-valued deep networks to efficient event-driven networks for image classification. Front. Neurosci. 11, 682. 10.3389/fnins.2017.00682 29375284 PMC5770641

[B100] Sabatier Q. Ieng S.-H. Benosman R. (2017). Asynchronous event-based fourier analysis. IEEE Trans. Image Process. 26, 2192–2202. 10.1109/TIP.2017.2661702 28186889

[B101] Safa A. Bourdoux A. Ocket I. Catthoor F. Gielen G. G. (2021a). A 2-μ j, 12-class, 91% accuracy spiking neural network approach for radar gesture recognition. arXiv preprint arXiv:2108.02669. 10.1109/TNNLS.2021.3109958 34520371

[B102] Safa A. Corradi F. Keuninckx L. Ocket I. Bourdoux A. Catthoor F.. (2021b). Improving the accuracy of spiking neural networks for radar gesture recognition through preprocessing. IEEE Trans. Neural Netw. Learn. Syst. 1–13. 10.1109/TNNLS.2021.3109958 34520371

[B103] Schmidt R. (1986). Multiple emitter location and signal parameter estimation. IEEE Trans. Antennas Propag. 34, 276–280.

[B104] Schuman C. D. Plank J. S. Bruer G. Anantharaj J. (2019). Non-traditional input encoding schemes for spiking neuromorphic systems, in 2019 International Joint Conference on Neural Networks (IJCNN) (Budapest: IEEE), 1–10.

[B105] Schumann O. Hahn M. Scheiner N. Weishaupt F. Tilly J. Dickmann J. Whler C. (2021). RadarScenes: A real-world radar point cloud data set for automotive applications, in 2021 IEEE 24th International Conference on Information Fusion (IEEE), 1–8. 10.23919/FUSION49465.2021.9627037

[B106] Schumann O. Wöhler C. Hahn M. Dickmann J. (2017). Comparison of random forest and long short-term memory network performances in classification tasks using radar, in 2017 Sensor Data Fusion: Trends, Solutions, Applications (SDF) (Bonn), 1–6.

[B107] Sengupta A. Ye Y. Wang R. Liu C. Roy K. (2019). Going deeper in spiking neural networks: vgg and residual architectures. Front. Neurosci. 13, 95. 10.3389/fnins.2019.00095 30899212 PMC6416793

[B108] Shalumov A. Halaly R. Tsur E. E. (2021). Lidar-driven spiking neural network for collision avoidance in autonomous driving. Bioinspir. Biomim. 16, 066016 10.1088/1748-3190/ac290c 34551395

[B109] Stuijt J. Sifalakis M. Yousefzadeh A. Corradi F. (2021). μbrain: an event-driven and fully synthesizable architecture for spiking neural networks. Front. Neurosci. 15, 538. 10.3389/fnins.2021.664208 34093116 PMC8170091

[B110] Sun S. Petropulu A. P. Poor H. V. (2020). Mimo radar for advanced driver-assistance systems and autonomous driving: advantages and challenges. IEEE Signal Process. Mag. 37, 98–117.

[B111] Thakur C. S. Molin J. L. Cauwenberghs G. Indiveri G. Kumar K. Qiao N.. (2018). Large-scale neuromorphic spiking array processors: a quest to mimic the brain. Front. Neurosci. 12, 891. 10.3389/fnins.2018.00891 30559644 PMC6287454

[B112] Theunissen F. E. Elie J. E. (2014). Neural processing of natural sounds. Nat. Rev. Neurosci. 15, 355–366. 10.1038/nrn3731 24840800

[B113] Thorpe S. Gautrais J. (1998). Rank order coding, in Computational Neuroscience (Boston, MA: Springer), 113–118.

[B114] Tsang I. J. Corradi F. Sifalakis M. Van Leekwijck W. Latré S. (2021). Radar-based hand gesture recognition using spiking neural networks. Electronics 10, 1405. 10.3390/electronics10121405

[B115] Viale A. Marchisio A. Martina M. Masera G. Shafique M. (2021). Carsnn: an efficient spiking neural network for event-based autonomous cars on the loihi neuromorphic research processor, in 2021 International Joint Conference on Neural Networks (IJCNN) (Shenzhen), 1–10.

[B116] Vogels T. P. Rajan K. Abbott L. F. (2005). Neural network dynamics. Annu. Rev. Neurosci. 28, 357–376. 10.1146/annurev.neuro.28.061604.135637 16022600

[B117] Wang S. Song J. Lien J. Poupyrev I. Hilliges O. (2016). Interacting with soli: Exploring fine-grained dynamic gesture recognition in the radio-frequency spectrum, in Proceedings of the 29th Annual Symposium on User Interface Software and Technology (New York, NY), 851–860.

[B118] Wu Y. Deng L. Li G. Zhu J. Shi L. (2018). Spatio-temporal backpropagation for training high-performance spiking neural networks. Front. Neurosci. 12, 331. 10.3389/fnins.2018.00331 29875621 PMC5974215

[B119] Wunderlich T. Kungl A. F. Müller E. Hartel A. Stradmann Y. Aamir S. A.. (2019). Demonstrating advantages of neuromorphic computation: a pilot study. Front. Neurosci. 13. 10.3389/fnins.2019.00260 30971881 PMC6444279

[B120] Yan Y. Stewart T. Choo X. Vogginger B. Partzsch J. Höppner S.. (2021). Comparing loihi with a spinnaker 2 prototype on low-latency keyword spotting and adaptive robotic control. Neuromorphic Comput. Eng. 1, 16. 10.1088/2634-4386/abf150

[B121] Yin B. Corradi F. Bohté S. M. (2021). Accurate and efficient time-domain classification with adaptive spiking recurrent neural networks. Nat. Mach. Intell. 3, 905–913. 10.1038/s42256-021-00397-w

[B122] Yin T. Zhou X. Krähenbühl P. (2020). Center-based 3d object detection and tracking. arXiv:2006.11275.

[B123] Yu F. Chen H. Wang X. Xian W. Chen Y. Liu F. Madhavan V. Darrell T. (2020). Bdd100k: A diverse driving dataset for heterogeneous multitask learning, in 2020 IEEE/CVF Conference on Computer Vision and Pattern Recognition (IEEE), 2633–2642. 10.1109/CVPR42600.2020

[B124] Zenke F. Ganguli S. (2018). Superspike: supervised learning in multilayer spiking neural networks. Neural Comput. 30, 1514–1541. 10.1162/neco_a_01086 29652587 PMC6118408

[B125] Zhou S. Chen Y. Li X. Sanyal A. (2020). Deep scnn-based real-time object detection for self-driving vehicles using lidar temporal data. IEEE Access 8, 76903–76912. 10.1109/ACCESS.2020.2990416

[B126] Zhou X. Wang D. Krähenbühl P. (2019). Objects as points. CoRR abs/1904.07850.

