# Supplementary material for: Automotive Radar Processing With Spiking Neural Networks: Concepts and Challenges
Source: Front Neurosci. 2022 Apr 1;16:851774. doi: 10.3389/fnins.2022.851774 (PMC9012531; doi:10.3389/fnins.2022.851774)
Supplement: Supplementary file 1 [file Data_Sheet_1.pdf]

Supplementary Material:
Automotive Radar Processing with Spiking Neural Networks: Concepts and Challenges

1 DETAILS ON SPIKING CFAR

1.1 Parameters of Conventional CFAR Algorithms

The parameters for the two conventional constant false alarm rate (CFAR) algorithms were chosen to allow a fair comparison of the spiking approaches: Both approaches use an array of 15×15 training cells, from which the inner 7×7 cells are discarded (guard cells), resulting in effective 176 training cells. For the cell-averaging CFAR (CA-CFAR) we chose the scale factor $\alpha = 5$ (cf. Equation (4) in main article) and the equivalent scaling factor $\alpha_c = 0.2$ for the ordered-statistic CFAR (OS-CFAR) (see López-Randulfe et al. (2021)). The parameter $k = 44$ for the OS-CFAR was selected such that both algorithms have approximately the same total number of CFAR detections on the considered dataset of 1000 frames (17115 for CA-CFAR respectively 17197 for OS-CFAR).

The conventional algorithms are applied to the signal amplitudes of the range-Doppler map from the CARRADA dataset. For this, the radar frames are converted from power in decibel to amplitudes. Furthermore, before applying the CFAR, we remove the static reflections from all the range-Doppler maps. Therefore, for each bin the median value is determined from the complete dataset and subtracted from the bin value. Especially, this removes static reflections at zero velocity and such reduces the number of CFAR detections originating fron non-radar targets (e.g. the road).

For cells under test located at the edges of the range-Doppler map, the window of training cells extends beyond the map. To cope with that, the original range-Doppler map is extended on all 4 sides as followed: The velocity dimension is wrapped around following the shape of a toroid. Concretely, the leftmost 7 columns are copied and shifted next to the right end of the map. Equivalently, the rightmost columns are copied to the left end. This is possible as the Doppler bins reflect the phase shift between chirps between $-\pi$ and π (See e.g. Gonzalez et al. (2021) for more details). The range dimension is expanded on each side by 7 rows with the mean amplitude value of the entire range-Doppler map.

1.2 Spiking CA-CFAR

1.2.1 Proof of equivalence to original algorithm

The spiking implementation of the CA-CFAR presented in Section 3.3.2 of the main article directly implements the CFAR condition $x_{\text{CUT}} > \alpha P_{\text{noise}}$, which can be derived as follows: The membrane potential v at time T is obtained by integrating the current I (Equation (11) of main article) over time:

$$v(T) = \int_0^T I(t)\mathrm{d}t \tag{S1}$$

$$= \int_0^T \sum_i \Theta(t - t_i) w_i \mathrm{d}t \tag{S2}$$

$$= \sum_i (T - t_i) w_i, \tag{S3}$$

where $\Theta(\cdot)$ denotes the Heaviside step function. Inserting the spike time definition for t_i from Equation (10) of the main article yields:

$$v(T) = \sum_i T(1 - \frac{x_{\max} - x_i}{x_{\max}}) w_i \tag{S4}$$

$$= \sum_i \frac{T}{x_{\max}} x_i \cdot w_i. \tag{S5}$$

The condition $v(T) > 0$ is equivalent to Equation (9) of the main article.

1.2.2 Spiking CA-CFAR Experiments

In Section 4.1 of the main article, the spiking CA-CFAR is evaluated with respect to the number of time steps and compared to the conventional CA-CFAR. For obtaining the spike times t_i from the input values $\hat{x}_i$ according to Equation (10) of the main article, the upper bound $\hat{x}_{\max}$ is set to the maximum value per range-Doppler map. After conversion, the spike times are discretized to multiples of T/N, where T is the total time and N the number of time steps. Three rounding schemes are considered: round down, round to nearest, and round up. Figure S1 shows a comparison of the different rounding approaches for the spiking CA-CFAR applied to 1000 range-Doppler frames from the CARRADA dataset and evaluated regarding sensitivity and precision for different number of time steps. When rounding down, there are no false positive detections such that the precision is always 1. Instead, there are many missed detections (false negatives) so that the sensitivity stays below 1 even for higher number of timestamps. When rounding to the nearest value, the sensitivity is high even for very few number of time steps, so there are hardly any false negatives. Instead, the precision is bad, as there are many false positives for low number of time steps. At around 300 time steps both indicators show close to perfect results. Rounding up has the worst performance: While the sensitivity is high all the time, there are many false positives even for 500 time steps.

1.3 Spiking OS-CFAR

The spiking OS-CFAR implements an ordered-statistics winner-takes-all spiking neural network (SNN), where the output neuron spikes if the central cell with value x_{CUT} spikes earlier than the k-th largest neighbour cell with value x_k (López-Randulfe et al., 2021). The translation from range-Doppler map values to input spike times is defined as

$$t(x_i) = -(t_{\max} - t_{\min}) \frac{x_i - x_{\min}}{x_{\max} - x_{\min}} + t_{\max}. \tag{S6}$$

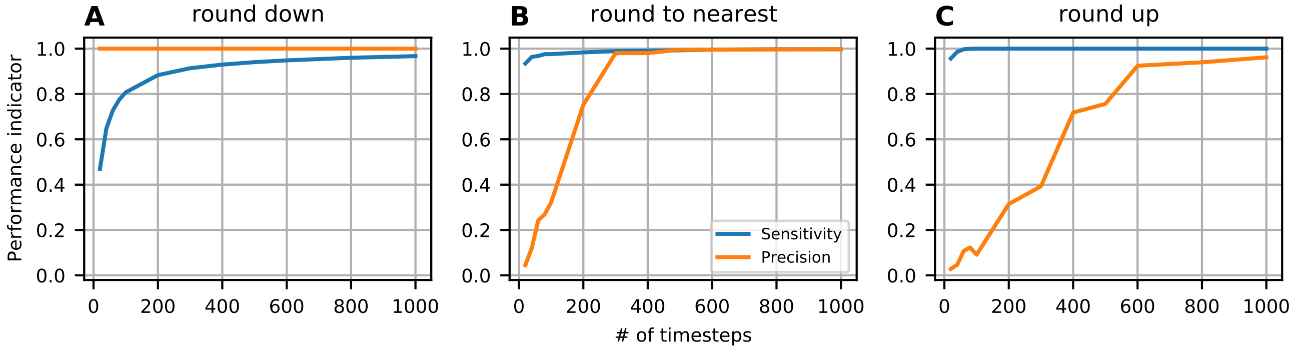

Figure S1. Comparison of rounding approaches for spiking CA-CFAR. **(A)** down rounding, **(B)** nearest rounding, **(C)** up rounding.

Here, $x_{\min}$ and $x_{\max}$ are boundaries containing all considered x_i values, while $t_{\min}$ and $t_{\max}$ are the first and last possible spike times which are typically set to 0 and the simulation time T. If $x_{\min}$ is additionally set to 0, then the conversion of spike times is identical to the CA-CFAR (Equation (10) in main article). Note that the spike time for the neuron representing the cell under test (CUT) considers the scaling factor α_c:

$$t_{\mathrm{CUT}} = t(\alpha_c x_{\mathrm{CUT}})\,. \tag{S7}$$

In simulations of the spiking OS-CFAR network the spike times are also discretized to multiples of the time step T/N. Namely, all spikes within the range $[nT/N, (n+1)T/N]$ are processed at the same time step n, which is equivalent to rounding down in the spiking CA-CFAR.

Three variants of the spiking OS-CFAR are considered that differ in the way how the spike times are generated:

1. **Amplitudes:** The amplitude values of the range-Doppler map are used to generate the input spike times according to equations (S6-S7). This variant represents the original approach from López-Randulfe et al. (2021).
2. **Power in decibel:** Here, power values in decibel are used instead of amplitudes to obtain the spike times. The scaling factor α_c is modified accordingly such that this variant is mathematically equivalent to the first one.
3. **Power in decibel + delay:** In addition to the use of power values in decibel for the spike time generation, a delay of $\tau_d = t_s/2$ is added to the spike times of the training cells, where t_s is the SNN simulation time step. While this variant is no longer identical to the original OS-CFAR, it shows the best results when the number of time steps is limited.

1.3.1 Spiking OS-CFAR Experiments

Here we evaluate three different variants of the spiking OS-CFAR algorithm regarding their performance in comparison with the traditional OS-CFAR algorithm. Figure S2A shows the results of the baseline variant with amplitudes as input: The precision yields 1 independent of the number of time steps as there are no false positive detections. Instead, the sensitivity starts at a very small value, then continuously increases with the number of time steps but even for 1000 time steps differs significantly from 1. The observed misdetections are cases where the converted spike time t_{CUT} of the CUT is lower than the spike time t_k of the k-th largest neighbour cell, but both spike times fall into the same time step. Then, the output

neuron does not fire a spike. Choosing a nearest rounding or ceiling approach for converting values to spike times would not affect the final outcome significantly, as the order of the values would stay unaltered and again t_{CUT} and t_k can be binned to the same time step.

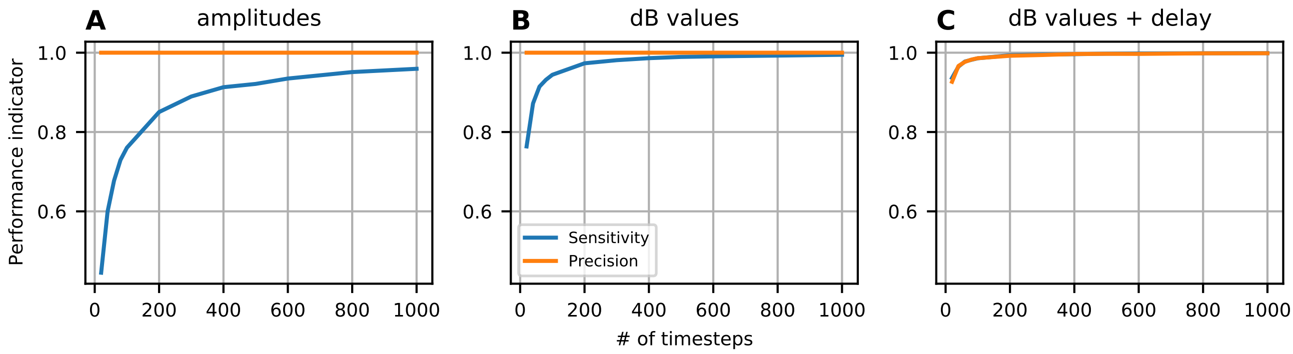

Figure S2. Comparison of the spiking OS-CFAR performance compared to the original algorithm. **(A)** Input data provided in amplitudes. **(B)** Input data converted to a logarithmic scale before feeding it to the algorithm. **(C)** In addition to **B**, the spikes coming from all neighbour cells are delayed by half time step.

The better results of the spiking CA-CFAR for few time steps, when compared to those of the spiking OS-CFAR, can be explained by looking at the distribution of data values in the range-Doppler maps. The two histograms in figure S3 show the values of an exemplary range-Doppler map from the CARRADA dataset (Ouaknine et al., 2021) for signal *amplitudes* and for signal *power in decibel*, respectively. The amplitude distribution is very narrow and mostly occupies a few bins while the dB histogram is much wider. As data points from the same bin are mapped to the same time step, the described cases of misdetections for the spiking OS-CFAR are more likely. On the other hand, the effect is smaller for the spiking cell-averaging CFAR with nearest rounding, as the discretization effect is averaged out by considering all training cells and not only the k-th largest value.

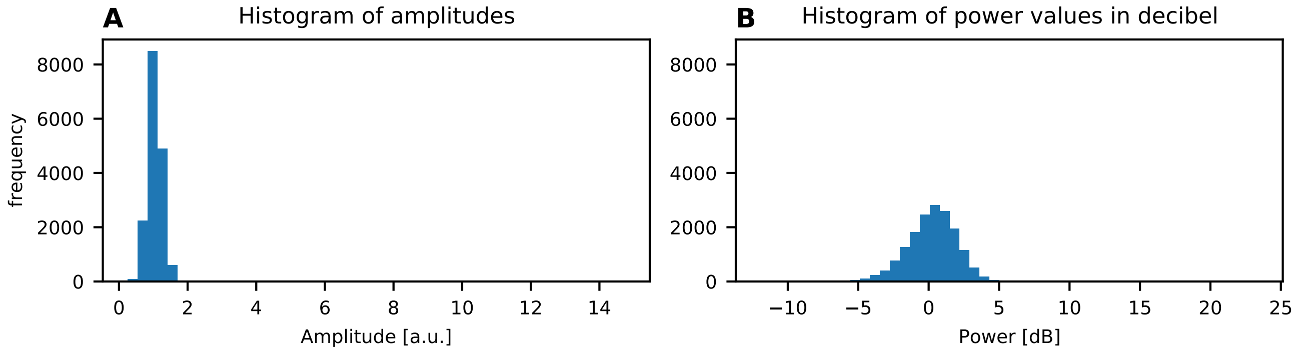

Figure S3. Histogram of data values of an exemplary range-Doppler frame: **A:** amplitude values, **B** power values in decibel. For both histograms, 50 bins of equal size were used between the minimum and maximum values in the data.

Two modifications are applied to improve the performance of the spiking OS-CFAR. First, we process the range-Doppler map values in dB power instead of amplitudes while adapting the scaling factor α_c accordingly. This does not change the outcome of the original OS-CFAR algorithm, as only the order of

values matters, which is maintained by the conversion from amplitudes to dB power. The result is shown in figure S2B: While the precision is always 1, the sensitivity is now much closer to 1 even for low number of time steps. Second, to further decrease the number of missing detections, we modify the incoming spikes from the neighbour cells by adding a delay of τ_d. This way, some of the false negative cases where t_{CUT} and t_k with $t_{\mathrm{CUT}} < t_k$ fall into the same time steps are eliminated increasing the sensitivity. On the other hand, the network will produce some false positives, since t_{CUT} will anticipate t_k in some cases where it should not. We have chosen a delay of $\tau_d = t_s/2$ based on empirical results, where t_s is the simulation time step. Both changes are represented in figure S4, and the improvement of this modification is depicted in figure S2C. Overall, the combination of processing input values in dB and adding a delay to the neighbour cells provides the best compromise regarding sensitivity and precision.

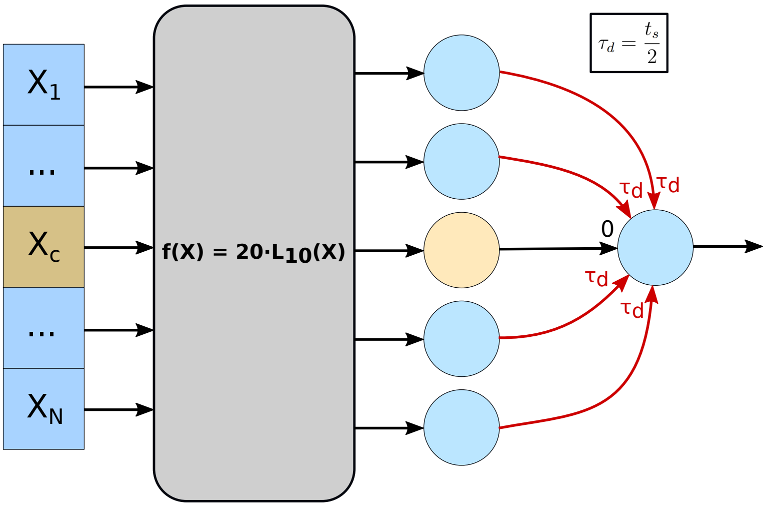

Figure S4. Diagram of the spiking OS-CFAR processing chain. The input in the left represents a window from the range-Doppler map centered around the cell under test X_C and containing N neighbour cells. The data is converted to a logarithmic scale and fed to the SNN described in the main article. The synaptic connections between the neighbour cells and the output neuron (in red) are modified with a time delay of $t_s/2$, where t_s is the simulation time step.

2 DETAILS ON TARGET CLASSIFICATION

2.1 Dataset Generation of Range-Doppler ROI sequences

For the SNN-based radar target classification in Section 4.2 of the main article a sub-dataset containing sequences of range-Doppler region of interests (ROIs) was extracted from the CARRADA dataset (Ouaknine et al., 2021) as follows: For each object within each range-Doppler frame the box annotations were used to calculate a center point. A statistical analysis on the overall annotated bounding boxes indicates that a region of 26 range bins and 20 Doppler bins covers over 90% of the bounding box area, thus a ROI with this constant size was chosen and the normalized data without static offset removal was taken around the calculated center point. The events were generated by comparing each data point to a constant threshold of 0.5. When the center point is located at the edge of the available dimensions, the ROI would extend across the original range-Doppler map: In the cases crossing the range boundary, it is forced to shift the ROI inside to ensure the data is all extracted from the RD pixels. For the Doppler boundary case, a Doppler padding scheme is applied similar to the CFAR data-preparation in section 1.1 due to the Doppler circularity of the range Doppler map. For each extracted ROI the location is compared to the

locations of the previous frame and in case of an overlap of the regions and a matching label it is assumed that the ROI belongs to the same object as in the previous frame. The result is a time-ordered sequence of ROIs of the same object. From this, shorter fundamental sequences of 8 ROI frames composed yielding 399 car-, 208 bicycle- and 323 pedestrian sequences. Each ROI appears only once, i.e., the sequences do not overlap, and remaining sequences with less than 8 frames are discarded. At last, the dataset is split into 80% train and 20% test data. All given accuracies are based on the test set by averaging 12 runs.

2.2 Details on SNN Model

The SNN using leaky integrate & fire (LIF) neurons is implemented in a discrete time domain similar to Bellec et al. (2020) defined by the following equations:

$$V[t_n] = \alpha_\tau V[t_{n-1}] + (1-\alpha_\tau)\, I[t_n] - I_{\text{reset}}. \tag{S8}$$

$$I_{\text{reset}} = Z[t_{n-1}]\,\theta. \tag{S9}$$

$$Z[t_n] = \begin{cases} 1, & \text{if } V[t_n] > \theta \\ 0, & \text{otherwise} \end{cases} \tag{S10}$$

$$I_i[t_n] = \sum_j W_{ij} Z_j[t_{n-1}] + \sum_k R_{ik} Z_k[t_{n-1}]. \tag{S11}$$

V defines the membrane potential, $\alpha_\tau = \exp\left(\frac{-1}{\tau}\right)$ is the decay factor depending on time constant τ. I_{reset} resets the membrane voltage by the threshold θ if the neuron spiked in the previous time step indicated by variable Z. The input current I_i to neuron i in (S11) combines the synaptic events from the previous and the recurrent layer.

2.3 Details on Network Models

All networks were implemented in TensorFlow (Abadi et al., 2015), the structure of the networks is illustrated in figure S5. Network **A** is a 2D-convolutional neural network (CNN) trained on ROIs of single frames without temporal dependency. The ReLU is used as nonlinearity in the convolutional and dense layers. This network provides a reference on the classification accuracy that can be achieved on a single frame. Networks **B** and **C** are the artificial neural network (ANN) and SNN that operate on extracted ROI sequences over multiple consecutive frames. Both networks share the same overall structure and layer sizes. The SNN uses LIF neurons instead of ReLUs in the convolutional layers, respectively LIF instead of LSTM units in the recurrent layer. The output layer of the SNN uses non-spiking integrator neurons as in Bellec et al. (2020). For all networks a learning rate of 0.001 over 100 epochs and the Adam optimizer is used. For SNN training a piecewise linear, triangle-like function is used as surrogate gradient during backpropagation through time (BPTT). The networks B and C are simulated for 8 time steps which corresponds to the number of frames in the ROI sequence.

REFERENCES

Abadi, M., Agarwal, A., Barham, P., Brevdo, E., Chen, Z., Citro, C., et al. (2015). TensorFlow: Large-scale machine learning on heterogeneous systems. Software available from tensorflow.org

**A**: CNN	**B**: ANN	**C**: SNN
Conv F:6 K:(3,3)	TimeDist(Conv) F:6 K:(3,3)	Conv-SNN F:6 K:(3,3)
MaxPool K:(2,2)	TimeDist(MaxPool) K:(2,2)	TimeDist(MaxPool) K:(2,2)
Conv F:16 K:(3,3)	TimeDist(Conv) F:16 K:(3,3)	Conv-SNN F:16 K:(3,3)
MaxPool K:(2,2)	TimeDist(MaxPool) K:(2,2)	TimeDist(MaxPool) K:(2,2)
Flatten	TimeDist(Flatten)	TimeDist(Flatten)
Dense N:30	LSTM N:32	LIF N:32
Dense N:30	Dense N:3	Integrator N:3
Dense N:3		

Figure S5. Network structures for object classification. K: kernel size, F: number of filters, N: number of neurons. The wrapper-statement TimeDist(...) is used to indicate that the wrapped operation is applied on each discrete time step.

Bellec, G., Scherr, F., Subramoney, A., Hajek, E., Salaj, D., Legenstein, R., et al. (2020). A solution to the learning dilemma for recurrent networks of spiking neurons. *Nature communications* 11, 1–15

Gonzalez, H. A., Liu, C., Vogginger, B., Kumaraveeran, P., and Mayr, C. G. (2021). Doppler disambiguation in mimo fmcw radars with binary phase modulation. *IET Radar, Sonar & Navigation* 15, 884–901. doi:https://doi.org/10.1049/rsn2.12063

López-Randulfe, J., Duswald, T., Bing, Z., and Knoll, A. (2021). Spiking neural network for fourier transform and object detection for automotive radar. *Frontiers in Neurorobotics* 15. doi:10.3389/fnbot.2021.688344

Ouaknine, A., Newson, A., Rebut, J., Tupin, F., and Pérez, P. (2021). Carrada dataset: Camera and automotive radar with range- angle- doppler annotations. In *2020 25th International Conference on Pattern Recognition (ICPR)*. 5068–5075. doi:10.1109/ICPR48806.2021.9413181
